# Guide to nonlinear potential estimates

**DOI:** 10.1007/s13373-013-0048-9

**Published:** 2014-01-09

**Authors:** Tuomo Kuusi, Giuseppe Mingione

**Affiliations:** 1 Aalto University Institute of Mathematics, P.O. Box 11100, 00076 Aalto, Finland; 2 Dipartimento di Matematica e Informatica, Università di Parma, Parco Area delle Scienze 53/a, Campus, 43124 Parma, Italy

## Abstract

One of the basic achievements in nonlinear potential theory is that the typical linear pointwise estimates via fundamental solutions find a precise analog in the case of nonlinear equations. We give a comprehensive account of this fact and prove new unifying families of potential estimates. We also describe new fine properties of solutions to measure data problems.

## A synopsis

The aim of this paper is to give a rather comprehensive introduction to nonlinear potential estimates, i.e., pointwise estimates for solutions to quasilinear, possibly degenerate elliptic equations via linear and nonlinear potentials. The paper contains both new and old results. They fall into two categories. The first consists of those results that have been proved elsewhere, and that are here given in different and/or streamlined version. The second contains new results that are presented for the first time. These fill some of the gaps that were making the current theory still somewhat incomplete.

To ease the reading, the already known results will be stated together with the reference to the corresponding original paper where they have appeared for the first time. The new ones will be presented pointing at the place of this paper where the proof can be found. In general, the first part of the paper is devoted to the presentation of the general setting, with the statements of the main theorems; this goes up to Sect. 8. The remaining parts are instead devoted to the proofs.

We shall start from a presentation of the classical results valid for linear elliptic equations in Sect. 2, and this will serve to give the general guideline to the topics we are going to cover in the nonlinear case. The first potential estimates for nonlinear equations will be introduced in Sect. 3. There the classical pointwise estimates will be presented. In the subsequent Sects. 4 and 5 we shall instead give a class of estimates aimed at unifying the theory. These allow to frame the classical pointwise potential inequalities in a more general setting, allowing for estimates of both size and oscillations of solutions and their derivatives, including fractional derivates. At this stage we shall use “intermediate” Wolff and Riesz potentials and various fractional maximal operators. Especially, we prove Theorem 10, whose role is to unify estimates for $u$ and $Du$ via a family of estimates for suitable fractional operators of $u$. We shall eventually add a few remarks on the case of equations with coefficients in Sect. 6. All the results up to Sect. 6 are presented for *energy solutions*, that is for solutions belonging to the natural energy space $W^{1,p}$ associated to the problems considered. In Sect. 7 we then turn to the case of general measure data problems, that is when *very weak solutions* come into the play. This will give us the opportunity, in Sect. 8, to present a few interesting consequences of potential estimates. Specifically, we shall prove two theorems about the possibility of describing the fine behaviour of solutions to nonlinear measure data problems and of their gradient via potentials. These theorems are the nonlinear analogs of classical results about fine properties of solutions to linear equations, which are usually derived via linear potential; see Remark 3 below. In Sect. 9 we then gather a series of regularity results that follow as a corollary of the theory presented. Some of these results are well-known and they are now framed in the general and unifying context of nonlinear potential estimates. The remaining sections are devoted to the proofs of the results introduced in the preceding ones, and their titles are self-explaining. In this paper we are not going to deal with parabolic problems, for which we refer to [42–44].

Before starting, we find useful to establish some notation. In what follows we denote by $c$ a general positive constant, possibly varying from line to line; special occurrences will be denoted by $c_1, c_2, {\bar{c}}_1, {\bar{c}}_2$ or the like. All these constants will always be *larger or equal than one*; moreover relevant dependencies on parameters will be emphasized using parentheses, i.e., $c_{1}\equiv c_1(n,p,\nu , L)$ means that $c_1$ depends only on $n,p,\nu , L$. We denote by

$$\begin{aligned} B(x,r)\equiv B_r(x):=\{\tilde{x} \in {\mathbb { R}}^n \, : \, |\tilde{x}- x|< r\} \end{aligned}$$

the open ball with center $x$ and radius $r>0$. When not important we shall omit denoting the center as follows: $B_r \equiv B(x,r)$. Moreover, with $B$ being a generic ball with radius $r$, we will denote by $\sigma B$ the ball concentric to $B$ having radius $\sigma r$, $\sigma >0$. Unless otherwise stated, different balls in the same context will have the same center. The symbol $\Omega$ will denote a bounded open subset of ${\mathbb { R}}^n$ and we shall always consider the case $n \ge 2$. With ${\mathcal { O}} \subset {\mathbb { R}}^{n}$ being a measurable subset with positive measure, and with $g :{\mathcal { O}} \rightarrow {\mathbb { R}}^{n}$, $n\ge 1$, being a measurable map, we shall denote byits integral average; here $|{\mathcal { O}}|$ denotes the Lebesgue measure of ${\mathcal { O}}$. The map $g$ will be typically a gradient. We shall denote

$$\begin{aligned} |g|(O):=\int _{\mathcal { O}} |g| \, d\tilde{x} \end{aligned}$$

therefore adopting a unified notation for both measures and integrals of $L^1$-maps. In the following, $\mu$ will always denote a Borel measure with finite total mass, which is initially defined on a certain open subset $\Omega \subset {\mathbb { R}}^n$. Since this will not affect the rest, with no loss of generality, all such measures will be considered as defined in the whole ${\mathbb { R}}^n$ so that $|\mu |({\mathbb { R}}^n) < \infty .$ We shall denote by ${\mathcal { M}}_\mathrm{b}$ the space of all such measures.

## From linear to nonlinear

When considering linear elliptic equations, a powerful tool for establishing the qualitative properties of solutions is given by representation formulas via fundamental solutions. Eventually, these lead to consider linear Riesz potentials and singular integrals. Let us recall the situation for the simplest example, which is of course given by the classical Poisson equation

1 $$\begin{aligned} -\triangle u =\mu . \end{aligned}$$

For the sake of exposition, we initially consider the last equation in the whole ${\mathbb { R}}^n$, with $n \ge 2$. Here $\mu$, which is eventually taken to be a measure, is again for simplicity assumed to be a smooth and compactly supported function, while $u$ is the unique solution which decays to zero at infinity. The point we are interested in now is that $u$ can be recovered via convolution with the so called fundamental solution

2 $$\begin{aligned} G(x,y) \approx \left\{ \begin{array}{l@{\quad }ll} |x-y|^{2-n}&{} \hbox { if } &{} n\ge 3\\ - \log \, |x-y|&{} \hbox { if } &{} n= 2 \end{array}\right. \end{aligned}$$

and this means that the following representation formula holds:

3 $$\begin{aligned} u(x) = \int _{{\mathbb { R}}^n} G(x,y)\, d\mu (y). \end{aligned}$$

The formula in the last display allows to shift the study of solutions to the analysis of a related integral operator. It is therefore time for the following:

### **Definition 1**

(*Riesz potentials*) Let $\alpha \in (0,n]$; the linear operator defined by

4 $$\begin{aligned} I_{\alpha }(\mu )(x):= \int _{{\mathbb { R}}^n} \frac{d\mu (\tilde{x})}{|x-\tilde{x}|^{n-\alpha }}, \end{aligned}$$

is called the $\alpha$-Riesz potential of $\mu$, where $\mu$ is a Borel measure defined on ${\mathbb { R}}^n$.

Now, (3) allows to conclude with the following pointwise potential estimates:

5 $$\begin{aligned} |u(x)|\lesssim I_{2}(|\mu |)(x)|\qquad \hbox { and}\qquad |Du(x)|\lesssim I_{1}(|\mu |)(x). \end{aligned}$$

The first inequality actually holds in the case $n\ge 3$; the second one follows differentiating (3). Since the behaviour of Riesz potentials with respect to various relevant function spaces is known (see for instance [1, 26]), using (5) allows to infer *size* properties of solutions from those of the relevant potentials, and in a sharp way. For instance, the following regularizing property of Riesz potentials is known for every $q>1$ and $\alpha >0$ such that $0< \alpha q< n$:

$$\begin{aligned} \Vert I_\alpha (\mu )\Vert _{L^{\frac{nq}{n-\alpha q}}({\mathbb { R}}^n)}\le c (n,\alpha , q)\Vert \mu \Vert _{L^{q}({\mathbb { R}}^n)}. \end{aligned}$$

As an immediate corollary, it follows for instance that $\mu \in L^{q}$ implies that $Du \in L^{nq/(n-q)}$ whenever $1 < q < n$. Moreover, estimates in further function spaces can be obtained as well, and these yield borderline regularity results. An instance is given by the following Lorentz spaces criterion:

6 $$\begin{aligned} \mu \in L(n,1) \Longrightarrow I_1(|\mu |) \in L^{\infty } \Longrightarrow Du \in L^{\infty }. \end{aligned}$$

We recall that the Lorentz space $L(q,\gamma )(\Omega )$, for $1\le q < \infty$ and $\gamma >0$, consists of those measurable maps $\mu$ satisfying the condition

7 $$\begin{aligned} \int _0^\infty \left( \lambda ^{q}|\{\tilde{x} \in \Omega \, : \, |\mu (\tilde{x})|>\lambda \}|\right) ^{\gamma /q} \, \frac{d\lambda }{\lambda } < \infty \end{aligned}$$

so that $\mu \in L(n,1)$ means in particular that

8 $$\begin{aligned} \int _0^\infty |\{\tilde{x} \in \Omega \, : \, |\mu (\tilde{x})|>\lambda \}|^{1/n} \, d\lambda < \infty . \end{aligned}$$

A point, which is less emphasised concerning estimates (5) is that (3) also allows to bound *oscillations* of the solution $u$ in terms of suitable Riesz potentials. This point actually turns out of be of primary interest for us here. To see this, by using the elementary inequality

$$\begin{aligned} \left| |x-\tilde{x}|^{2-n}-|y-\tilde{x}|^{2-n}\right| \le c(n) \left| |x-\tilde{x}|^{2-n-\alpha }+|y-\tilde{x}|^{2-n-\alpha }\right| |x-y|^\alpha , \end{aligned}$$

which is valid whenever $x,y,\tilde{x} \in {\mathbb { R}}^n$, we get the new representation formula

9 $$\begin{aligned} |u(x)-u(y)| \le c\left[ I_{2-\alpha }(|\mu |)(x)+I_{2-\alpha }(|\mu |)(y)\right] |x-y|^\alpha \,, \quad 0 \le \alpha \le 1. \end{aligned}$$

The previous estimate allows to infer, again via potentials, information on the solutions in function spaces measuring smoothness rather than size. For instance, the Hölder continuity criterion

$$\begin{aligned} I_{2-\alpha }(|\mu |) \in L^{\infty } \Longrightarrow u \in C^{0,\alpha } \end{aligned}$$

is immediate. Again, prescribing that $I_{2-\alpha }(|\mu |) \in L^{q}$ implies that $u$ belongs to the so called Calderón space $C^{\alpha }_q$; see Definition 2 below, and [17, 40], for a discussion. In a similar way one gets estimates for the gradient

10 $$\begin{aligned} |Du(x)-Du(y)| \le c\left[ I_{1-\alpha }(|\mu |)(x)+I_{1-\alpha }(|\mu |)(y)\right] |x-y|^\alpha \,, \quad 0 \le \alpha < 1.\qquad \end{aligned}$$

As a matter of fact, formulas (9)–(10) can be read as nonlocal, fractional derivatives versions of the classical estimates in (5). This is clarified by the following definition:

### **Definition 2**

(*Calderón spaces*) Let $\alpha \in (0,1]$, $q \ge 1$, and let $\Omega \subset {\mathbb { R}}^n$ be an open subset. A measurable function $v$, finite a.e. in $\Omega$, belongs to the Calderón space $C^{\alpha }_q(\Omega )$ if and only if there exists a nonnegative function $m\in L^q(\Omega )$ such that

11 $$\begin{aligned} |v(x)-v(y)|\le [m(x)+ m(y)]|x-y|^\alpha \end{aligned}$$

holds for almost every couple $(x,y)\in \Omega \times \Omega$.

This is just another way to say that $v$ has “fractional derivatives”. The advantage is that nonlocality is reduced to a minimal status: only two points are considered in (11). Moreover, when $\Omega$ is a suitably regular domain, Calderón spaces are closely related to the usual fractional Sobolev spaces $W^{\alpha , q}$; see [17]. The function $m$ plays in fact the role of a fractional derivative of $v$ of order $\alpha$ in the $L^q$-sense. Definition 2 is implicit in the work of DeVore and Sharpley [17], where the authors fix the canonical choice $m=M^{\#}_{\alpha }(v)$ that is indeed always possible in (11) (see Proposition 1 below). The symbol $M^{\#}_{\alpha }(v)$ denotes the standard fractional sharp maximal operator introduced in the following (take $\Omega ={\mathbb { R}}^n$ and $R= \infty$):

### **Definition 3**

(*Fractional sharp maximal operator*) Let $\beta \in [0,1]$, $x \in \Omega$ and $R \le \mathrm{dist} (x, \partial \Omega )$, and let $f\in L^1(\Omega )$; the function defined byis called the restricted (centered) sharp fractional maximal function of $f$.

Notice that for $\beta =0$ the previous definition gives back the classical sharp maximal operator of Fefferman and Stein (in the restricted version), which is in fact denoted by $M_{R}^{\#} (f)$. Definition 2 and the interpretation of $m$ as the $\alpha$-order fractional derivative of $v$ allow to read the inequalities in (9)–(10) as a way to bound fractional derivatives of $u$ via intermediate Riesz potentials. We may express this concept in the following imprecise yet suggestive way:

12 $$\begin{aligned} |\partial ^\alpha u(x)|\lesssim I_{2-\alpha }(|\mu |)(x), \quad 0 \le \alpha < 2. \end{aligned}$$

Needless to say, that last formula has only a symbolic meaning. At this point it is clear that the classical estimates (5) embed in the family of interpolating estimates (12) as particular borderline cases corresponding to the choices $\alpha =0$ and $\alpha =1$, respectively. In turn, the estimates in (12) provides a unifying approach to the regularity of solutions to (1). They allow to get results both in spaces aimed at measuring the size of functions and in those measuring smoothness.

One of the aims of this paper is to show that completely similar estimates actually hold for a large class of quasilinear, possibly degenerate equations. A potential theory which is completely analogous to the linear one can be constructed in the nonlinear case too.

## Basic nonlinear estimates

The main question is now whether and in which sense estimates like (5) and (12) extend to the case of solutions to quasilinear elliptic equations of the type

13 $$\begin{aligned} -\hbox {div}\, a(Du)=\mu \quad \hbox { in } \ \Omega . \end{aligned}$$

In the most general case, the right hand side $\mu$ is a Borel measure with finite total mass in ${\mathbb { R}}^n$, i.e. $\mu \in {\mathcal {M}}_\mathrm{b}$. The one in (13) is a nonlinear version of the Poisson equation when the assumptions

14 $$\begin{aligned} \nu \, \mathrm{Id}\le \partial a(z) \le L \mathrm{Id} \end{aligned}$$

are considered. The numbers $0< \nu \le L$ provide bounds for the lowest and highest eigenvalue of the matrix $\partial a (\cdot )$, respectively ($\nu =L=1$ gives the case of the Laplacean operator). We also want to consider cases where Eq. (13) might be degenerate, thereby examining for instance the so called $p$-Laplacean operator:

15 $$\begin{aligned} -\hbox {div}\, (|Du|^{p-2}Du)=\mu . \end{aligned}$$

In order to catch the essential properties of the equation in (15), we shall consider general vector fields $a:{\mathbb { R}}^n \rightarrow {\mathbb { R}}^n$. These are assumed to be $C^1$-regular and to satisfy the following *growth and ellipticity assumptions*:

16 $$\begin{aligned} \left\{ \begin{array}{l} |a(z)|+|\partial a(z)|(|z|^2+s^2)^{1/2} \le L(|z|^2+s^2)^{(p-1)/2} \\ \nu (|z|^2+s^2)^{(p-2)/2}|\lambda |^{2} \le \langle \partial a(z)\lambda , \lambda \rangle \\ p \ge 2 \end{array} \right. \end{aligned}$$

whenever $z, \lambda \in {\mathbb { R}}^n$. The case $p=2$ gives back (14). We remark that we confine ourselves to the case $p \ge 2$ as we are mainly interested to present the main ideas in the most accessible way; for results in the case $p\le 2$ we refer to [22, 38, 40, 58]. In the rest of the paper $\nu , L$ are fixed parameters whose role is to establish the rate of ellipticity of the vector field $a(\cdot )$. The role of $s\ge 0$ is more peculiar. This parameter serves to distinguish the degenerate ($s=0$) from the nondegenerate one ($s>0$). In this last case a model is given, by for instance taking $s=1$, by the nondegenerate equation $p$-Laplacean type equation (see [49])

$$\begin{aligned} -\hbox {div}\, ((1+|Du|^2)^{(p-2)/2}Du)=\mu . \end{aligned}$$

Sometimes we shall also consider equations with measurable coefficients, that is when the vector field $a(\cdot )$ exhibits an explicit dependence on the variable $x$, i.e.,

17 $$\begin{aligned} -\hbox {div}\, a(x,Du)=\mu . \end{aligned}$$

In this case, we shall use a set of assumptions which is weaker than (16), namely, we shall consider a Carathéodory vector field $a:\Omega \times {\mathbb { R}}^n \rightarrow {\mathbb { R}}^n$ such that

18 $$\begin{aligned} \left\{ \begin{array}{l} |a(x,z)| \le L(|z|^2+s^2)^{(p-1)/2} \\ \nu (|z_1|^2+|z_2|^2+s^2)^{(p-2)/2}|z_1-z_2|^{2} \le \langle a(x,z_1)-a(x,z_2), z_1-z_2 \rangle \\ p \ge 2 \end{array} \right. \end{aligned}$$

are satisfied whenever $z_1,z_2$, $x \in \Omega$. These assumptions are, up to adjusting the constants $\nu , L$ in a universal way, implied by those in (16). Results for the case when $x \rightarrow a (x, \cdot )$ is more regular and satisfy (16) uniformly with respect to $x \in \Omega$ are described in Sect. 6 below.

Our primary emphasis will be on a priori estimates. This means that, according to a scheme which is typical in regularity theory, we shall mainly confine ourselves to present results in form of a priori estimates for more regular solutions and data. We are therefore most of the times considering weak energy solutions to (17), that is functions $u \in W^{1, p}(\Omega )$ satisfying

19 $$\begin{aligned} \int _{\Omega } \langle a( \tilde{x},Du),D\varphi \rangle \, d \tilde{x} =\int _{\Omega } \varphi \, d \mu \end{aligned}$$

for every choice of $\varphi \in C^{\infty }(\Omega )$ with compact support in $\Omega$. Here we are still assuming that $\mu$ is a Borel measure with finite mass, but we could assume that $\mu \in C^{\infty }$ as well, stating the same results involving only the total variation of $\mu$, considered as a measure. Results for the original context would then follow via approximation. Such an approach is obviously restrictive when considering general measure data problems. Indeed, observe for instance that due to the monotonicity properties of the operator assumed in (16), considering a solution that belongs to the space $W^{1,p}(\Omega )$ automatically implies that the measure $\mu$ belongs to the dual $W^{-1,p'}$, which is certainly not the case for any measure when $p \le n$. As a matter of fact, in general, distributional solutions to measure data problems do not belong to the natural energy space $W^{1,p}(\Omega )$ and for this reasons they are called *very weak solutions*. Treating the case of general measures needs then greater care, already in specifying the notion of solution one is dealing with. We are briefly discussing these aspects in Sects. 7 and 14.4 below, where we shall see how to extend the results presented for energy solutions to the case of general solutions to measure data problems.

Since we are going to deal with local results, we shall use in a standard way the truncated version of the classical Riesz potentials.

### **Definition 4**

(*Truncated Riesz potentials*) Let $\mu$ be Borel measure with finite total mass on ${\mathbb { R}}^n$; the (truncated) Riesz potential is defined by

$$\begin{aligned} \mathbf{I}_{\beta }^\mu (x,R):= \int _0^R \frac{|\mu |(B(x,\varrho ))}{\varrho ^{n-\beta }}\, \frac{d\varrho }{\varrho }\,, \qquad \beta >0, \end{aligned}$$

whenever $$x \in {\mathbb { R}}^n$$ and $$0< R \le \infty $$.

The word truncated refers to the inequality $\mathbf{I}_{\beta }^\mu (x,R) \le c(n) I_\beta (|\mu |)(x)$. Now, let us go back to estimates in (5), and let us notice that these cannot hold when $p \not = 2$. Indeed, they clearly do not respect the homogeneity properties of the equation. To see this, we consider a non-null solution to $\hbox {div}\, (|Du|^{p-2}Du)= \mu$ with $p>2$, then, for $\gamma >0$ we have

$$\begin{aligned} \tilde{u}: =\gamma ^{1/(p-1)} u \Longrightarrow \hbox {div}\, (|D\tilde{u}|^{p-2}D\tilde{u})= \gamma \mu := \tilde{\mu }. \end{aligned}$$

Assuming now that the first estimate in (5) would hold, this would yield

$$\begin{aligned} |\tilde{u}(x)|\lesssim I_{2}(|\tilde{\mu }|)(x), \end{aligned}$$

that is, recalling the definitions of $\tilde{u}$, $\tilde{\mu }$ and $I_{2}$,

$$\begin{aligned} |u(x)|\lesssim \gamma ^{(p-2)/(p-1)}I_{2}(\mu )(x). \end{aligned}$$

Upon letting $\gamma \rightarrow 0$ and using that $p>2$, we would then conclude with $|u(x)|=0$ whenever $I_{2}(\mu )(x)$ is finite. A similar argument—letting $\gamma \rightarrow \infty$—also gives that estimate as in (5) cannot hold in the case $p<2$. In order to overcome this point one is led to consider a new family of potentials. They are obtained by, in some sense, incorporating in Riesz potentials the scaling properties of the $p$-Laplacean equation.

### **Definition 5**

(*Wolff potentials*) Let $\mu$ be Borel measure with finite total mass on ${\mathbb { R}}^n$; the nonlinear Wolff potential is defined by

$$\begin{aligned} \mathbf{W}^{\mu }_{\beta , p}(x,R):= \int _0^R \left( \frac{|\mu |(B(x,\varrho ))}{\varrho ^{n-\beta p}}\right) ^{1/(p-1)}\, \frac{d\varrho }{\varrho }, \quad \beta >0 \end{aligned}$$

whenever $$x \in {\mathbb { R}}^n$$ and $$0< R \le \infty $$.

Wolff potentials, that despite their name were first considered and studied in [28], reduce to Riesz potentials when $p=2$, i.e. $\mathbf{I}_{\beta }^\mu \equiv \mathbf{W}_{\beta /2,2}^\mu$. They play a crucial role in nonlinear potential theory and in the description of the fine properties of solutions to nonlinear equations in divergence form [2, 3, 28, 29, 32, 59, 60].

An important fact about Wolff potentials is that their behaviour can be in several aspects recovered from that of Riesz potentials via so called Havin–Maz’ya potentials $\mathbf{V}_{\beta ,p}(|\mu |)(x)$. Indeed, following inequality holds provided $p\beta < n$:

$$\begin{aligned} \mathbf{W}^{\mu }_{\beta , p}(x,\infty ) \lesssim I_{\beta }\left\{ \left[ I_{\beta }(|\mu |)\right] ^{1/(p-1)}\right\} (x)=: \mathbf{V}_{\beta ,p}(|\mu |)(x). \end{aligned}$$

The above estimate allows to derive, in a sharp way, almost all types of local estimates starting by the properties of the Riesz potentials, whose action in several function spaces is in fact known; for this we refer to [28, 29].

As first shown in the fundamental works of Kilpeläinen and Malý [37, 38] for the case of nonnegative measures, a neat analog of the first estimate in (5) holds using Wolff potentials. Later on, a new and interesting proof has been offered Trudinger and Wang [65, 66], and this allows to cover the case of general subelliptic operators. Yet different proofs can be found in [39], and in [21], where an approach covering the case of general signed measures has been developed. The final outcome is summarized in the following:

### **Theorem 1**

[21, 37, 38, 65] Let $u \in W^{1,p}(\Omega )$ be a weak solution to the equation with measurable coefficients (17) under the assumptions (18). There exists a constant $c \equiv c (n,p,\nu ,L)$ such that the pointwise estimate20holds whenever $B(x,R)\subset \Omega$ and the right hand side is finite. Moreover, if

21 $$\begin{aligned} \lim _{R\rightarrow 0}\mathbf{W}_{1,p}^{\mu }(x, R)=0 \quad \hbox { locally uniformly in} \ \Omega \ \hbox { w.r.t. } \ x \end{aligned}$$

then $u$ is continuous in $\Omega$.

A sketchy proof of this theorem is proposed in Sect. 18 below; extensions to more general operators have been given in [50, 53]. If $p=2$, then $\mathbf{W}_{1,p}^\mu \equiv \mathbf{I}_{2}^{\mu }$ and we retrieve a local analog of the first estimate in (5). A remarkable point here is that estimate (20) is sharp, and the nonlinear potential $\mathbf{W}_{1,p}^\mu$ cannot be replaced by any other smaller potential. This is in fact reported in the following:

### **Theorem 2**

[37, 38] Let $u \in W^{1,p}(\Omega )$ be a nonnegative weak solution to the Eq. (13) under the assumptions (18), where $\mu$ is a positive measure and $s=0$. There exists a constant $c \equiv c (n,p,\nu ,L)$ such that the following pointwise estimate holds whenever $B(x,2R)\subset \Omega$ and the Wolff potential is finite:

$$\begin{aligned} c^{-1}\mathbf{W}_{1,p}^\mu (x,R) \le u(x) \le c\mathbf{W}_{1,p}^\mu (x,2R)+ c\inf _{B(x,R)} u. \end{aligned}$$

The possibility of extending pointwise potential estimates to the gradient of solutions has remained an open and discussed issue since the paper [38]. The answer came only recently and here we consider the case of equations of the type (13). The first result in this direction is contained in [57] for the case $p=2$, that is when assumptions (14) are in force. In [57] it is indeed proved the following analog of the second estimate in (5):22For the case $p > 2$ a first result has been given in [21], where the following, apparently natural estimate, has been proved to hold at every Lebesgue point $x$ of $Du$, under assumptions (16):23The previous estimate seems to put a final word on the problem of a sharp analog of the second estimate in (5) since the orthodoxy of nonlinear potential theory prescribes that Wolff potentials replace Riesz potentials everywhere when $p\not =2$. Surprisingly enough, in [41] we have shown that this is not the case. Using Wolff potentials is necessary only when estimating solutions, while it is not when passing to their gradients. In fact the following holds:

### **Theorem 3**

[41] Let $u \in W^{1,p}(\Omega )$ be a solution to the Eq. (13), under the assumptions (16). There exists a constant $c \equiv c (n,p,\nu ,L)$ such that the Riesz potential estimate24holds whenever $B(x,R)\subset \Omega$ and the right hand side is finite. Moreover, if

25 $$\begin{aligned} \lim _{R\rightarrow 0}\mathbf{I}_1^{\mu }(x, R)=0 \ \hbox { locally uniformly in } \ \Omega \ \hbox { w.r.t. } \ x \end{aligned}$$

then $Du$ is continuous in $\Omega$.

The proof of Theorem 3 will be presented in Sect. 15 below, where it will be obtained as a corollary of more general potential estimates. An extension of the previous result to a class of general operators including the $p$-Laplacean has been recently given by Baroni in [5].

### *Remark 1*

An obvious ambiguity arises in the statements of Theorems 1–3 when saying that the related estimates hold whenever the right hand side is finite. This might appear obvious. Another point is that estimates in Theorems 1–3 are stated for every $x$, while both $u(x)$ and $Du(x)$ are only defined if $x$ is Lebesgue point, where the so called precise representative can be defined. Both ambiguities are clarified in Sect. 8. There we prove that, both for $u$ and $Du$, the set of Lebesgue points coincides with the one for which inequalities (20) and (24) feature a finite right hand side, respectively.

Observe that estimate (24) obviously improves the one in (23) as, if $p\ge 2$ then $1/(p-1)\le 1$, and therefore

26 $$\begin{aligned} \mathbf{I}_1^{\mu }(x,R)&= \int _0^R \frac{|\mu |(B(x_0,\varrho ))}{\varrho ^{n-1}}\, \frac{d\varrho }{\varrho } \nonumber \\&\lesssim \sum _{i=0}^\infty \frac{|\mu |(B(x,R/2^{i}))}{(R/2^{i})^{n-1}} \nonumber \\&\lesssim \left[ \sum _{i=0}^\infty \left( \frac{|\mu |(B(x,R/2^{i}))}{(R/2^{i})^{n-1}}\right) ^{1/(p-1)}\right] ^{p-1}\nonumber \\&\lesssim \left[ \int _0^{2R} \left( \frac{|\mu |(B(x_0,\varrho ))}{\varrho ^{n-1}}\right) ^{1/(p-1)}\, \frac{d\varrho }{\varrho } \right] ^{p-1} \nonumber \\&= \left[ \mathbf{W}_{1/p,p}^{\mu }(x,2R)\right] ^{p-1}. \end{aligned}$$

The implications of Theorem 3 are rather surprising: when switching to the gradient, estimates *linearize* and they reduce to the ones already available for the Poisson equation. The nonlinear, possibly degenerate character of the equations considered plays no role here. In particular, the regularity theory of equations as (13) reduces to that of the Poisson equation up to the $C^1$-level. Several facts typical of the linear theory can now be reproduced verbatim. For instance, for solutions $u \in W^{1,p}({\mathbb { R}}^n)$ to (13) defined in the whole ${\mathbb { R}}^n$, the classical estimate via classical Riesz potentials

$$\begin{aligned} |Du(x)|^{p-1} \le c\int _{{\mathbb { R}}^n} \frac{d|\mu |(y)}{|x-y|^{n-1}} \end{aligned}$$

follows immediately. For a relevant connection between Theorem 3 and fundamental solutions to general nonlinear measure data problems we refer to Sect. 7.1 below. Another very precise analogy with the classical linear theory is a striking nonlinear extension of a fundamental theorem of Stein [63]. This claims that if $v \in W^{1,1}$ is a Sobolev function defined in ${\mathbb { R}}^n$ with $n \ge 2$, then

27 $$\begin{aligned} Dv \in L(n,1) \, \Longrightarrow \ v \ \hbox { is continuous}. \end{aligned}$$

This can be regarded as the limiting case of Sobolev–Morrey embedding theorem that reads $Dv \in L^{n+\varepsilon } \ \Longrightarrow \ v \in C^{0,\varepsilon /(n+\varepsilon )}$ whenever $\varepsilon >0$. Note indeed that $L^{n+\varepsilon }\subset L(n,1)\subset L^n$ for every $\varepsilon > 0$ with all the inclusions being strict. Another way to state Stein’s theorem concerns the regularity of solutions $u :\Omega \rightarrow {\mathbb { R}}^N$ to the Laplacean system, and reads

$$\begin{aligned} \hbox {div}\, Du = \triangle u \in L(n,1) \ \Longrightarrow \ Du \ \hbox { is continuous}. \end{aligned}$$

This follows by (27) and classical Calderón–Zygmund theory. The point is now that if $\mu \in L(n,1)$, then a computation involving the definition of norm in Lorentz spaces (see [23, 41] for discussion) implies that (25) takes place, and therefore we conclude with the following:

### **Theorem 4**

(Nonlinear Stein theorem [41]) Let $u \in W^{1,p}(\Omega )$ be a solution to the Eq. (13), under the assumptions (16) and such that $\mu \in L(n,1)$ locally in $\Omega$. Then $Du$ is continuous in $\Omega$.

See [12] for a global Lipschitz bound. Without appealing to potentials, but by using different means, the result of the previous theorem also holds for systems.

### **Theorem 5**

(Vectorial nonlinear Stein theorem [45]) Let $u \in W^{1,p}(\Omega , {\mathbb { R}}^N)$, $N\ge 1$, be a vector valued solution to the $p$-Laplacean system

$$\begin{aligned} -\triangle _p u =F. \end{aligned}$$

Assume that the components of the vector field $F :\Omega \rightarrow {\mathbb { R}}^N$ locally belong to the space $L(n,1)$. Then $Du$ is continuous in $\Omega$.

Finally, let us mention that starting from the techniques developed for the last two theorems, similar results can be proved in the case of fully nonlinear equations [15].

## Universal potential estimates

The results of this section have a double aim. On the one hand, they show that estimates (9)–(10) have analogs for nonlinear equations. On the other hand, they show that those in (20), (23) and (24) are actually special cases of a more general class of nonlinear potentials estimates. These, in turn, allow to recover in an optimal way all the basic regularity properties of solutions in terms of the regularity of the assigned datum $\mu$. The range of the results implied by such estimates is of course limited by the regularity theory of homogeneous equations as

28 $$\begin{aligned} \hbox {div}\, a(x,Dw)=0. \end{aligned}$$

Therefore we first recall what is the maximal regularity of solutions to equations as in (28) and in (29) below. In turn, this dramatically changes according to the smoothness assumed on the partial map $x \rightarrow a(x, \cdot )$. When measurability is considered, very low degree of regularity is expected and solutions are just Hölder continuous for some exponent. When instead dependence on $x$ becomes more regular then higher regularity follows. In this case, again for sake of simplicity, and since this does not affect the expositions of the main ideas, we shall confine ourselves to the case of equations with no coefficients as

29 $$\begin{aligned} \hbox {div}\, a(Dw)=0. \end{aligned}$$

The first result we present upgrades estimate (20) to low order fractional derivatives, and allows to give a sharp formulation of the classical De Giorgi’s theory via nonlinear Wolff potential estimates. De Giorgi’s theory for equations with measurable provides the existence of a *universal Hölder continuity exponent* $\alpha _m \in (0,1)$, depending only on $n,p,\nu , L$, such that

30 $$\begin{aligned} w \in C^{0,\alpha }_\mathrm{loc}(\Omega ) \qquad \hbox {for every } \ \alpha < \alpha _m \end{aligned}$$

and31The previous estimate holds whenever $x,y \in B_{R/2}$ and $B_R \subset \Omega$, for a constant depending only on $n,p,\nu , L$ and $\alpha$. The exponent $\alpha _m$ can be thought as the *maximal Hölder regularity exponent* associated to the vector field $a(\cdot )$. It is universal in the sense that it is independent of $a(\cdot )$ and of the particular solution considered, but just depends only on $n,p,\nu , L$. For more precise information on $\alpha _m$ see Theorem 18 and Remark 6 below. It then holds the following:

### **Theorem 6**

(De Giorgi’s theory via potentials [40]) Let $u \in W^{1,p}(\Omega )$ be a weak solution to the equation with measurable coefficients (17) under assumptions (18). Let $B_R \subset \Omega$ be such that $x,y\in B_{R/2}$; then32holds provided the right hand side is finite and $0 \le \alpha < \alpha _m$, where the exponent $\alpha _m$ has been defined in (30)–(31). Moreover, whenever $\tilde{\alpha }\in [0, \alpha _m)$ is fixed, the dependence of the constant $c$ is uniform for $\alpha \in [0,\tilde{\alpha }]$, in the sense that $c$ depends only on $n,p,\nu , L$ and $\tilde{\alpha }$.

Notice that when $\mu \equiv 0$ estimates (31) and (33) coincide. In general, counterexamples show that in (30) $\alpha _m \rightarrow 0$ when $L/\nu \rightarrow \infty$. This prevents estimate (33) to hold in general for the full range $\alpha \in [0,1)$ when $x \rightarrow a(x, \cdot )$ is measurable. Things change when the dependence on $x$ is more regular. For instance, when considering Eq. (29), estimate (31) holds for $\alpha =1$. In this case the validity of (32) extends to the whole interval $\alpha \in [0,1]$. Moreover, estimates (20) and (23) can be uniformly obtained as limiting endpoint cases of (32) for $\alpha =0$ and $\alpha =1$, respectively. In fact, it holds the following:

### **Theorem 7**

(Uniform Wolff potential estimate [40]) Let $u \in W^{1,p}(\Omega )$ be a weak solution to the Eq. (13) under assumptions (16). Let $B_R \subset \Omega$ be such that $x,y\in B_{R/2}$; then estimate (32) holds for $\alpha \in [0,1]$, whenever the right hand side is finite. The constant $c$ depends only on $n,p,\nu , L$ but is otherwise independent of $\alpha$.

Estimate (32) does not catch up, when $\alpha \rightarrow 1$, the optimal gradient bound in (24). There is a natural reason for such a lack of endpoint property: it catches the other optimal bound (20) as $\alpha \rightarrow 0$, and the two cases involve different potentials. In fact, the change in the nature of the estimates when passing from (20) to (24) requires another theorem, parallel to Theorem 7.

### **Theorem 8**

(Uniform Riesz potential estimate) Let $u \in W^{1,p}(\Omega )$ be a weak solution to the Eq. (13) under assumptions (16). Let $B_R \subset \Omega$ be such that $x,y\in B_{R/4}$; then33holds provided the right hand side is finite and $0< \alpha \le 1$. Moreover, whenever $\tilde{\alpha }\in (0,1]$ is fixed, the dependence of the constant $c$ is uniform for $\alpha \in [\tilde{\alpha },1]$ as $c$ depends only $n,p,\nu , L$ and $\tilde{\alpha }$.

The previous result is here presented for the first time and for the proof we refer to Sect. 5 below. There Theorem 8 is actually obtained as a corollary of a more general estimate for certain maximal operators of $Du$. From the discussion made there it will be clear that the points $x,y$ are Lebesgue points of $u$ when the right hand side in (33) is finite; compare with Remark 1. Theorems 6 and 8 provide altogether the optimal analog of estimate (9) and in fact they both unify with Theorem 7 when $p=2$, when Wolff and Riesz potentials do coincide. We remark that is also possible to quantify the blow-up of the constant $c$ in (33) as $\alpha \rightarrow 0$; see Remark 7 below.

We now examine the situation for the gradient, giving an estimate which is again bound to give back (10) when $p=2$. As already done before Theorem 6, we recall the basic information about the maximal regularity of solutions to homogeneous equations as in (29). These in turn involve the fundamentals of the regularity theory of the $p$-Laplacean equations and can be summarised by saying that there exists a positive exponent $\alpha _M \in (0,1)$, depending only on $n,p,\nu$ and $L$, such that whenever $x,y \in B_{R/2}$,

34 $$\begin{aligned} Dw \in C^{0,\alpha }_\mathrm{loc}(\Omega ) \quad \hbox { for every } \ \alpha < \alpha _M \end{aligned}$$

and35hold *for any local solution* $w$ to (29). The constant $c$ depends only on $n,p,\nu , L$ and $\alpha$. For estimate (35) and the exponent $\alpha _M$ see Theorem 17 and Remark 5 below. The result in (34) finds its origins in the basic work of Uraltseva [67] while further approaches can be found in [18, 24, 46, 51]. We remark that all these results are again based on the groundbreaking work of De Giorgi [16]. The exponent $\alpha _M$ is in general strictly less than one (see again [67]); while lower bounds on $\alpha _M$ can be obtained by tracking the constant dependence in the various proofs, its precise (optimal) value is not known. An optimal lower bound for $\alpha _M$ is conjectured to be $1/3$, according to the regularity exhibited by the solutions to the so called $\infty$-Laplacean equation [4]. By taking (34) into account we have the following nonlinear version of estimate (10):

### **Theorem 9**

(Uniform Wolff potential estimate) Let $u \in W^{1,p}(\Omega )$ be a weak solution to the Eq. (13) under assumptions (16). Let $B_R \subset \Omega$ be such that $x,y\in B_{R/4}$; then36holds provided the right hand side is finite and

37 $$\begin{aligned} 0\le \alpha <\min \{1/(p-1), \alpha _M\}. \end{aligned}$$

The exponent $\alpha _M$ has been defined in (34). Moreover, whenever $0 \le \tilde{\alpha }< \min \{1/(p-1), \alpha _M\}$ is fixed, the dependence of the constant $c$ is uniform for $\alpha \in [0,\tilde{\alpha }]$, in the sense that $c$ depends only $n,p,\nu , L$ and $\tilde{\alpha }$.

The proof of this theorem will be presented in Sect. 16 below. Notice that the limitation in (37) makes the statement of Theorem 9 consistent with the definition of Wolff potential given in Definition 5, where it must be $\beta >0$.

## Maximal-potential estimates

Let us now explain Theorem 8 proceeding for some while with a few purely heuristic arguments. By considering the function $m$ appearing in (11) as a fractional derivative of order $\alpha$, we can express the content of Theorem 8 in the following way:

38 $$\begin{aligned} |\partial ^\alpha u(x)|^{p-1}\lesssim I_{p-\alpha (p-1)}(|\mu |)(x),\quad 0 < \alpha \le 1 \end{aligned}$$

thereby obtaining a nonlinear version of (12). The singularity of the case $\alpha =0$ essentially stems from the fact that Wolff potentials come then into the play when considering pointwise estimates for $u$. Let us proceed heuristically and see why Riesz potentials intervene as long as fractional derivatives are considered. We rewrite Eq. (15) as the decoupled system

$$\begin{aligned} \left\{ \begin{array}{l} -\hbox {div}\, H=\mu \\ H = |Du|^{p-2}Du. \end{array}\right. \end{aligned}$$

This viewpoint tells us that an equation as in (15), which is commonly seen as *a nonlinear equation in the gradient*, can be also seen as linear equation in a *nonlinear vector field of the gradient*, that is $H$. The analysis of equations as $-\hbox {div}\, H=\mu$, being $f \rightarrow \hbox {div}\, f$ a differential operator of order one, typically involves estimates of $H$ via operators as $\mathbf{I}_1^\mu$. In turn this explains the appearance of estimates as in (24). The reader will immediately recognize that no similar argument applies to $u$, as the equation depends directly on $Du$ and not $u$. This is ultimately the reason for the appearance of Wolff potentials when deriving pointwise for $u$ as (20). Let us now go to intermediate derivatives and let us believe that fractional derivatives $\partial ^\alpha u$ can be controlled by in turn controlling quantities as

39 $$\begin{aligned} \frac{|u-(u)_{B_R}|}{R^\alpha } \approx R^{1-\alpha }|Du|\approx |\partial ^\alpha u| \end{aligned}$$

on every possible ball $B_R$. Using a typical dimension analysis viewpoint, the appearance in the estimates of a multiplicative factor as $R^{-\beta }$ amounts to consider a corresponding derivative of order $\beta$ in the estimates. Now, let us rewrite Eq. (15) as

$$\begin{aligned} R^{(\alpha -1)(p-1)}\hbox {div}\, (|R^{1-\alpha }Du|^{p-2}R^{1-\alpha }Du) = \mu \end{aligned}$$

and note that in this scheme of reasoning the operator $f \rightarrow R^{(\alpha -1)(p-1)}\hbox {div}\, f$ has now order $p-\alpha (p-1)$. Its inversion should therefore involve potentials as $\mathbf{I}_{p-\alpha (p-1)}^\mu$. At this point, recalling the identification (39) leads to conclude with an estimate with the dimensional meaning of (38). The point is now to make everything rigorous. A natural way to make the identification in (39) is to use a nonlocal operator as for instance a fractional maximal operator. It is therefore time for the following:

### **Definition 6**

(*Fractional maximal operator*) Let $\beta \in [0,n]$, $x \in \Omega$ and $R < \mathrm{dist} (x, \partial \Omega )$, and let $f$ be an $L^1(\Omega )$-function or a measure with finite mass; the function defined by

$$\begin{aligned} M_{\beta , R} (f)(x):= \sup _{0 < r \le R} \, r^{\beta }\frac{|f|(B(x,r))}{|B(x,r)|} \end{aligned}$$

is called the restricted (centered) fractional $\beta$ maximal function of $f$.

Using fractional maximal operators allows to get Riesz potential estimates which are uniform in the whole range $[0,1]$, and that provide a rigorous interpretation of previous heuristic arguments. The outcome is summarized in the following theorem, which appears here for the first time. The proof is contained in Sect. 12 below (see Sect. 14.4 for the case of SOLA).

### **Theorem 10**

(Uniform maximal-potential estimates) Let $u \in W^{1,p}(\Omega )$ be a weak solution to the Eq. (13) under assumptions (16). Then for every ball $B(x,R) \subset \Omega$ the following estimate:40holds uniformly in $\alpha \in [0,1]$, with $c \equiv c(n,p,\nu , L)$.

The maximal operators of Definition 3 naturally connect to those presented in Definition 6 via Poincaré inequality

41 $$\begin{aligned} M_{\alpha , R}^{\#} (u)(x) \le cM_{1-\alpha ,R}(Du)(x),\quad \alpha \in [0,1] \end{aligned}$$

so that a uniform estimate on $M_{\alpha , R}^{\#} (u)$ follows appealing to Theorem 10, that is42We yet remark that in the estimate in the last display the constant $c$ is independent of $\alpha$, and in fact the estimate holds uniformly for $\alpha \in [0,1]$.

The turning point to pointwise estimates is now given by the fact that the sharp maximal operator $M_{\alpha , R}^{\#} (u)(x)$ controls the pointwise behaviour of $u$ provided $\alpha >0$. This fact is expressed in the following Proposition, a first form of which is present in [17]. It in turn relies on some original arguments of Campanato [10].

### **Proposition 1**

Let $f \in L^1(B_{8R/5})$; for every $\alpha \in (0,1]$ the inequality

43 $$\begin{aligned} |f(x)-f(y)|\le \frac{c}{\alpha }\left[ M^{\#}_{\alpha ,R}(f)(x)+M^{\#}_{\alpha ,R}(f)(y)\right] |x-y|^{\alpha } \end{aligned}$$

holds whenever $x,y \in B_{2R/5}$, for a constant $c$ depending only on $n$. More precisely, $x$ and $y$ are Lebesgue points of $f$ whenever $M^{\#}_{\alpha ,R}(f)(x)$ and $M^{\#}_{\alpha ,R}(f)(y)$ are finite, respectively. Therefore, whenever the right hand side in (43) is finite, the values of $f$ are defined as follows:

$$\begin{aligned} f(x):= \lim _{\varrho \rightarrow 0} (f)_{B(x,\varrho )} \quad \hbox {and} \quad f(y):=\lim _{\varrho \rightarrow 0} (f)_{B(y,\varrho )}. \end{aligned}$$

Proposition 1 allows to obtain Theorem 8 as a corollary of Theorem 10 and in particular of (42); see Sect. 12.7 below for the proofs, including the one of Proposition 1. We believe that Theorem 10 helps to understand the peculiar nature of the case $\alpha =0$ and the occurrence of Wolff potentials instead of Riesz potentials. Indeed, an $L^\infty$-bound on the sharp maximal operator is essentially equivalent to require that $u$ belongs to the space BMO (introduced in [34], see (68) below). On the other hand, a real $L^\infty$-bound on $u$ itself requires an $L^\infty$-bound on $\mathbf{W}_{1,p}^\mu$. This situation looks completely natural when considering typical examples of functions lying in BMO${\setminus } L^\infty$, as for instance $\log \, |x|$. The $\log$-scale is indeed the one making a difference between a potentials as $\mathbf{W}_{1,p}^\mu$ and $\mathbf{I}_1^\mu$. Taking for instance

$$\begin{aligned} \mu (x)= \frac{1}{|x|^{p}\log ^{\beta } \, |x| } \end{aligned}$$

we see that $\mathbf{I}_{p}^\mu (0,1) < \infty$ provided $\beta >1$ while $\mathbf{W}_{1,p}^\mu (0,1)< \infty$ provided $\beta > p-1$. In the next section we shall in fact describe a few additional results showing that also the case $\alpha =1$ reveals to be special when using maximal operators instead of potentials.

### Non-endpoint maximal estimates

An interesting fact concerning the two cases $\alpha =0,1$ of (40) is that when looking for bounds on intermediate derivatives, potentials can be replaced by maximal operators. These give in fact smaller quantities. Maximal operators, as we shall see, allow to replace conditions typically given in Lebesgue or Lorentz spaces, with weaker conditions formulated in terms of Marcinkiewicz spaces. Let us recall the following, elementary inequality which holds for $\gamma \in (0,1)$

$$\begin{aligned} M_{\beta ,\gamma R}(\mu )(x)\le c(n,\gamma , \beta ) \mathbf{I}_{\beta }^{\mu }(x,R), \end{aligned}$$

see for instance [40, Lemma 4.1]. Then we have the following:

#### **Theorem 11**

(Intermediate maximal estimates [40]) Let $u \in W^{1,p}(\Omega )$ be a weak solution to (13) under the assumptions (16). Let $B_R \subset \Omega$ be a ball centred at $x$; then the estimate44holds uniformly in $\alpha \in [0, \tilde{\alpha }]$, whenever $0<\tilde{\alpha }<1$. The constant $c$ depends only on $n,p,\nu , L, \tilde{\alpha }$.

As for gradient oscillations, we instead have the following result, whose proof is contained in Sect. 13 below.

#### **Theorem 12**

(Gradient sharp maximal estimate [40]) Let $u \in W^{1,p}(\Omega )$ be a weak solution to (13) under the assumptions (16). Let $B_R \subset \Omega$ be a ball centered at $x$; then the following estimate:holds uniformly in $\alpha \in [0, \tilde{\alpha }]$ whenever

$$\begin{aligned} 0\le \tilde{\alpha }<\min \{1/(p-1), \alpha _M\}, \end{aligned}$$

for a constant $c$ depending only on $n,p,\nu , L,\tilde{\alpha }$. The exponent $\alpha _M$ has been introduced in (34)–(35).

## Equations with coefficients

Getting nonlinear potential estimates catching regularity beyond Theorem 6 for equations as in (17), necessitates to assume more regularity on the partial map $x \mapsto a(x, \cdot )$. Keep in mind the discussion before Theorem 6. We therefore define the following averaged vector field:45whenever $B(x,r) \subset \Omega$, and then the averaged (and renormalized) modulus of continuity of $x \mapsto a(x, \cdot )$ as follows:46When considering equations of the type

47 $$\begin{aligned} -\hbox {div}\, (c(x)\tilde{a}(Du))=\mu \end{aligned}$$

the definition in (46) gives something which is comparable to the usual modulus of continuity of the function $c(\cdot )$. We then have

### **Theorem 13**

Let $u \in W^{1,p}(\Omega )$ be a weak solution to (17), with assumptions (16) (uniformly) verified by the partial map $z \rightarrow a(\cdot , z)$ for every $x \in \Omega$. Then

- [40] For every $\tilde{\alpha }\in (0,1)$ there exists a number $\delta \in (0,1)$, depending only on $n,p,\nu , L, \tilde{\alpha }$, such that
  48 $$\begin{aligned} \limsup _{\varrho \rightarrow 0}\, \omega (\varrho )\le \delta \end{aligned}$$
  implies the validity of estimate (32) whenever $\alpha \in [0,\alpha _m)$. In particular, if the limit in (48) is zero estimate (32) holds for every $\alpha < 1$
- [6] If the partial map $x \rightarrow a(x, \cdot )$ is Dini-continuous, i.e.
  49 $$\begin{aligned} \int _0 \omega (\varrho )\, \frac{d \varrho }{\varrho } < \infty , \end{aligned}$$
  then estimates (33) and (40) continue to hold, for $\alpha \in [\tilde{\alpha }, 1]$ and $\alpha \in [0 , 1]$, respectively
- [40] If the partial map $x \rightarrow a(x, \cdot )$ is Hölder continuous with exponent $\tilde{\alpha }< \min \{1/(p-1), \alpha _M\}$ in the sense that
  50 $$\begin{aligned} \sup _r \int _0^r \frac{ [\omega (\varrho )]^{2/p}}{\varrho ^{\tilde{\alpha }}}\, \frac{d \varrho }{\varrho } < \infty , \end{aligned}$$
  then estimate (36) holds whenever $\alpha < \tilde{\alpha }$.

In all three cases the constants involved in the estimates additionally depend on the quantities appearing in (48)–(50).

Let us now briefly comment on the assumptions made in the last theorem, remarking that all of them are essentially necessary. Assumption (48) allows to catch the case in which, when referring to Eq. (47), the function $c(\cdot )$ is not even continuous, but of class VMO or BMO with small seminorm. This reconnects the theory presented here to the classical Calderón–Zygmund theory, where VMO-regularity of coefficients is required to establish integrability estimates related to estimate (32); see for instance [9]. Assumption (149) is necessary too. Indeed, estimate (33) implies gradient boundedness when $\mu$ is good enough, while Dini continuity of coefficients is known to be necessary to get Lipschitz regularity of solutions; see [33]. The proof of the second statement in the last theorem has the techniques developed for Theorem 10 as starting point, and will appear in the forthcoming paper [6]. Finally, assumption (50) seems to be natural in view of the usual Schauder theory. This prescribes that, in order to have Hölder continuity of the gradient, the Hölder continuity of $x \mapsto a(x, \cdot )$ is necessary.

## Interlude on measure data problems

When looking at equations as in (17) we have considered distributional solutions lying in $W^{1,p}$. This choice is actually aimed at simplifying the presentation in a way that emphasises the results obtained in the form of a priori estimates. Dealing with $W^{1,p}$-solutions is anyway very much restrictive in view of the fact that, typically, distributional solutions to measure data problems do not enjoy this regularity. A typical instance is the so called *nonlinear Green’s function*

51 $$\begin{aligned} G_p(x) \approx \left\{ \begin{array}{l@{\quad }ll} \left( |x|^{\frac{p-n}{p-1}}-1\right) &{} \hbox { if } &{} 1< p \not =n\\ \log \ |x|&{} \hbox { if } &{} p= n \end{array}\right. \end{aligned}$$

which solves, in the sense of distributions, the problem

52 $$\begin{aligned} \left\{ \begin{array}{ll} -\triangle _p u=\delta &{} \quad \hbox {in } B(0,1)\\ u= 0&{}\quad \hbox {on } \partial B(0,1). \end{array}\right. \end{aligned}$$

In the right hand side of (52)$_1$ there appears the Dirac measure $\delta$ charging the origin. A straightforward calculation reveals that $G_p \not \in W^{1,p}$ while it holds that

53 $$\begin{aligned} |DG_p|^{p-1} \in {\mathcal {M}}^{n/(n-1)} \Longleftrightarrow |\{ |DG_p|^{p-1} > \lambda \}| \lesssim \lambda ^{-n/(n-1)}. \end{aligned}$$

We recall here that the Marcinkiewicz space ${\mathcal {M}}^q(\Omega )$, for $1 \le q < \infty$, is the set of all measurable maps $\mu$ satisfying the condition

54 $$\begin{aligned} \sup _{\lambda \ge 0} \, \lambda ^{q}|\{x\, : |\mu |(x)> \lambda \, \}|< \infty . \end{aligned}$$

The above discussion now motivates the following:

### **Definition 7**

(*Very weak solutions*) A function $u \in W^{1,1}_\mathrm{loc}(\Omega )$ is called a very weak solution $u$ to the Eq. (17) in $\Omega$ if $a(x,Du) \in L^{1}_\mathrm{loc}(\Omega ,{\mathbb { R}}^n)$ and (19) holds for every $\varphi \in C^{\infty }_c(\Omega )$.

The terminology “very weak” is used to emphasize the fact that such solutions do not belong, in general, to the natural Sobolev space $W^{1,p}(\Omega )$, i.e. they are not energy solutions. Note that, when considering assumptions (18), in order to guarantee that $a(x,Du) \in L^{1}(\Omega ,{\mathbb { R}}^n)$ it suffices, for instance, to have $Du \in W^{1,p-1}(\Omega )$. Definition 7, not surprisingly, poses problems. For instance, very weak solutions may exist beside usual energy solutions [61], and are therefore not in general unique. As a matter of fact one of the basic open issues of the theory of measure data problems is to find a function class where to solve in a unique way Dirichlet problems of the type

55 $$\begin{aligned} \left\{ \begin{array}{ll} -\hbox {div}\ a(x,Du)=\mu &{} \quad \hbox {in } \Omega \\ u= 0&{}\quad \hbox {on } \partial \Omega , \end{array}\right. \end{aligned}$$

with $\mu$ being in the most general case a (signed) Borel measure with finite total mass. We will not pursue this matter here, rather referring to [14, 36] for a more comprehensive discussion. Here we are interested in a class of solutions which we regard to be very natural (see in fact the equivalence results obtained in [36]) and for which all the potential estimates described in this paper continue to hold. This is the class of SOLA (Solutions Obtained by Limits of Approximations), which is introduced in the

### **Definition 8**

(*SOLA*) A function $u \in W^{1,1}_\mathrm{loc}(\Omega )$ is a SOLA to the Eq. (17) iff is a very weak solution and there exists a sequence of local energy solutions $\{u_k\} \subset W^{1,p}_\mathrm{loc}(\Omega )$ to the equations

$$\begin{aligned} -\hbox {div}\, a(x,Du_k)=\mu _k, \end{aligned}$$

such that $u_k \rightarrow u$ locally in $W^{1,p-1}(\Omega )$.

SOLA are special because they are selected via an approximation procedure using more regular energy solutions, and thereby they inherit a few of their basic properties. For instance: their precise representative is defined out of a null $p$-capacity set, exactly as $W^{1,p}$-functions; see Theorem 16 below. Let us recall how to build a SOLA to (55), following [8]. One considers solutions $u_k \in W^{1,p}_0(\Omega )$ to regularised problems

$$\begin{aligned} \left\{ \begin{array}{ll} -\hbox {div}\ a(x,Du_k)=\mu _k &{} \quad \hbox {in } \Omega \\ u_k= 0&{}\quad \hbox {on } \partial \Omega . \end{array}\right. \end{aligned}$$

The right hand sides data $\mu _k := \mu * \phi _k \in C^\infty$ are canonically obtained by smoothing $\mu$ via convolution with a sequence of smooth standard, smooth mollifiers $\{\phi _k\}$. This approach leads to the existence of a very weak solution $u \in W^{1,p-1}_0(\Omega )$ such that $u_k \rightarrow u$ in $W^{1,p-1}_0(\Omega )$ (up to a not relabeled subsequence). SOLA are not known to be unique, except in a few special cases (for instance when $\mu \in L^1$). See [8, 13, 14, 36] for a larger discussion. We summarise the basic existence and regularity results available for SOLA in the following:

### **Theorem 14**

[8, 55] Under the assumptions (18) with $p \le n$, there exists a SOLA $u \in W^{1,p-1}_0(\Omega )$ to (55). Moreover, every SOLA $u \in W^{1,1}_\mathrm{loc}(\Omega )$ to (17) is such that

56 $$\begin{aligned} u \in W^{1,q}_\mathrm{loc}(\Omega )\quad \hbox {for every }\quad q < n(p-1)/(n-1). \end{aligned}$$

In the case of equations of the type (13) under assumptions (16), we also have

57 $$\begin{aligned} Du \in W^{(1-\varepsilon )/(p-1), p-1}_\mathrm{loc} (\Omega )\quad \hbox {for every}\quad \varepsilon \in (0,1). \end{aligned}$$

A preliminary integrability result was obtained for a different kind of solutions in the pioneering paper of Lindqvist [48]. The restriction $p\le n$ is aimed at focusing on the case of SOLA, since when $p>n$ then $\mu$ belongs to the dual of $W^{1,p}$ and it is possible to consider standard energy solutions. In display (57) a fractional Sobolev space appears. The meaning is actually that

$$\begin{aligned} \int _{A} \int _{A} \frac{|Du(x)-Du(y)|^{p-1}}{|x-y|^{n+1-\varepsilon }}\, d x \, dy < \infty \qquad \hbox {for every}\ \ \varepsilon \in (0,1) \end{aligned}$$

holds whenever $A \Subset \Omega$. We also remark that, in fact, it can be proved that every SOLA $u$ is such that

58 $$\begin{aligned} |Du|^{p-1} \in {\mathcal {M}}^{n/(n-1)}(\Omega ). \end{aligned}$$

This means that every SOLA exhibits exactly the same integrability of the nonlinear Green’s function $G_p$ described in (53) (see [7, 20, 55]). Both the results in (56) and (57) are sharp, as follows again considering $G_p$, which can be in fact proved to be the only SOLA to the problem in (52). This last fact follows combining the results in [35, 62]. As mentioned above, by mean of approximations arguments, *all the potential estimate in this paper continue to hold for any SOLA* to (55). More details are in Sect. 14.4 below, where we in particular discuss the possibility of characterizing the Lebesgue points of SOLA via linear and nonlinear potentials.

### Potential estimates and fundamental solutions

To check to which extent estimates (20) and (24) replace the usual linear representation formulas in the nonlinear case, the best thing to do is to see how they reproduce the behaviour of the nonlinear fundamental solution $G_p$. We already know the optimality of estimate (20) from Theorem 2. We therefore concentrate on (24) and show that it actually reverses when considering the nonlinear Green’s function $G_p$. This means that for $x\in B(0,1){{\setminus }}\{0\}$ we have59with $R=2|x|$, and for a constant $c$ depending only on $n$ and $p$. Indeed by coarea formula we haveOn the other hand, as $\delta (B(x,\varrho ))=0$ for $\varrho \le R/2$ and $\delta (B(x,\varrho ))=1$ otherwise, we also have

$$\begin{aligned} \mathbf{I}_{1}^{\delta }(x,R) = \int _{R/2}^{R} \frac{d \varrho }{\varrho ^n}= \frac{2^n-1}{n-1}R^{1-n} \le c(n,p) |DG_p(x)|^{p-1}, \end{aligned}$$

so that (59) follows combining the last two inequalities.

## Fine properties of solutions via potentials

We have seen that linear and nonlinear potentials locally control the behaviour of solutions. It is at this point not surprising to discover that potentials also control their so called fine properties. In this respect we present two theorems. The former is concerned with the pointwise behaviour of gradients of SOLA, and in particular with their Lebesgue points. This result employs Riesz potentials. The latter in instead concerned with Lebesgue points of solutions and uses Wolff potentials.

### **Theorem 15**

Theorem 3 continues to hold whenever $u \in W^{1,p-1}(\Omega )$ is a SOLA to (13). Moreover, the condition

60 $$\begin{aligned} \mathbf{I}_{1}^\mu (x,R) < \infty \end{aligned}$$

implies that the following limit exists and therefore defines the precise representative of $Du$ at the point $x$:

61 $$\begin{aligned} \lim _{\varrho \rightarrow 0}\, (Du)_{B(x,\varrho )}=:Du(x). \end{aligned}$$

The proof of this last theorem is included in Sect. 17 below. The analogous statement for solutions is the following:

### **Theorem 16**

Theorem 1 continues to hold whenever $u \in W^{1,p-1}(\Omega )$ is a SOLA to (17). Moreover, the condition

62 $$\begin{aligned} \mathbf{W}_{1,p}^\mu (x,R) < \infty \end{aligned}$$

implies that the following limit exists and therefore defines the precise representative of $u$ at the point $x$:

63 $$\begin{aligned} \lim _{\varrho \rightarrow 0}\, (u)_{B(x,\varrho )}=:u(x). \end{aligned}$$

In particular, the set of non-Lebesgue points of $u$ has $p$-capacity zero.

We notice that the assertion about the $p$-capacity follows directly from the fact that set of points where the Wolff potential $\mathbf{W}_{1,p}^\mu$ blows-up has zero $p$-capacity (see for instance [30]).

### *Remark 2*

(Hausdorff dimension of singular sets of SOLA) Theorem 16 allows to define SOLA—which are initially defined only almost everywhere via convergence—outside a singular set (i.e. set of non-Lebesgue points) of Hausdorff dimension not larger than $n-p$, when $p \le n$. This establishes a connection with another class of solutions to measure data problem. These are called $p$-superharmonic functions and are defined in the case the measure $\mu$ is nonnegative; see [30, 38]. For such solutions every point is a Lebesgue point, by construction. The connection is now given by the fact that, in view of [36], every SOLA has a superharmonic representative (that is, they coincide almost everywhere) whenever the measure is nonnegative. In the case of Theorem 15 we instead can conclude that the Hausdorff dimension of the singular set of $Du$ is not larger than $n-1$. The last estimate follows on the other hand also by (57). Indeed, the Hausdorff dimension of the set of non-Lebesgue points of a general $W^{s,\gamma }$-map, with $s\gamma <n$, has Hausdorff dimension not larger that $n-s\gamma$; see [54].

### *Remark 3*

(Analogies with linear potential theory) Theorems 15 and 16 can be also considered as analogs of classical facts in linear potential theory, concerning the pointwise behaviour of solutions to the Poisson equation $-\triangle u=\mu$. As usual, in the linear case they follow from explicit representation formulas and abstract analysis of potentials. Here they are replaced by nonlinear potential estimates. Indeed, define

$$\begin{aligned} f(x):= (G_{\alpha }*g)(x) := \int _{{\mathbb { R}}^n} G_{\alpha }(x-y)g(y)\,dy, \end{aligned}$$

where $g \in L^q({\mathbb { R}}^n)$ with $q>1$. Here $G_\alpha$ denotes the $\alpha$-Bessel potential, that is defined via

$$\begin{aligned} G_{\alpha } := {\mathcal { F}}^{-1}\left( (1+|\xi |^2)^{-\alpha /2}\right) , \end{aligned}$$

where ${\mathcal { F}}$ denotes the Fourier transform and $\alpha \in (0,n)$. Then the limit

$$\begin{aligned} \lim _{\varrho \rightarrow 0}\, (f)_{B(x,\varrho )}=:f(x) \end{aligned}$$

exists—and thereby defines the precise representative of $f$—up to a set of zero $(\alpha , q)$-capacity. For these facts we refer to [3], and in particular to [3, Proposition 6.1.3]

## Regularity and corollaries

The estimates presented up to now allow to give a comprehensive and unified picture of the regularity results available for quasilinear equations. We will now briefly describe a few consequences of such estimates. First of all, let us recall that combining Theorem 11 and Proposition 1 yields the following pointwise inequality:64that holds whenever $x,y \in B_{R/4}$, $B_R \subset \Omega$, $\alpha \in (0,1)$, where $c\equiv c(n,p,\nu , L, \alpha )$. Notice here that the blow-up of $c$ takes place as soon as $\alpha$ approaches $0$ or $1$. In a completely similar way, by Theorem 12 and again Proposition 1, we then have that the inequality65holds with $x,y,r,B_R$ and $c$ as above, but for $\alpha \in (0,\alpha _M)$. This time the constant $c$ blows-up when $\alpha \rightarrow 0$ or $\alpha \rightarrow \alpha _M$. We are now interested in seeing how the results presented up to now, including estimates (64)–(65), imply regularity of solutions in various relevant function spaces. Let us briefly recall that the Morrey space $L^{q,\theta }(\Omega )$ for $q \ge 1$ and $0 \le \theta \le n$, is defined by requiring that its member $f \in L^{q}(\Omega )$ satisfies

66 $$\begin{aligned} \sup _{B_\varrho \subset \Omega } \varrho ^{\theta -n}\int _{B_{\varrho }}|f|^q\, d \tilde{x} < \infty , \end{aligned}$$

while their local version is defined in the usual way. We refer to [1, 56] for more on Morrey spaces and their basic properties with related references. We now have the following, comprehensive:

### **Corollary 1**

Let $u \in W^{1,p}(\Omega )$ be a weak solution—or a SOLA—to the Eq. (13) under assumptions (16). Then, with all the spaces being meant in their local versions, we have that

- (C1) if $\alpha \in (0,1)$ and $q > n(p-1)/[n-p+\alpha (p-1)]$, then
  $$\begin{aligned} \mu \in L^{\frac{nq}{n(p-1)+[p-\alpha (p-1)]q}} \Longrightarrow u \in C^{\alpha }_q \end{aligned}$$
  (estimate (64))
- (C2) if $$p< n$$, then
  $$\begin{aligned} \mu \in {\mathcal { M}}^{n/p}\Longrightarrow |\mu |(B_\varrho )\lesssim \varrho ^{n-p} \Longrightarrow u \in \hbox {BMO} \end{aligned}$$
  and
  $$\begin{aligned} p=n \Longrightarrow u \in \hbox {BMO} \end{aligned}$$
  (Theorem 11 with $$\alpha =0)$$
- (C3) $$\mathbf{W}_{1,p}^\mu \in L^{\infty } \Longrightarrow u \in L^\infty $$ (Theorem 1)
- (C4) if $$ p < n$$, then $$\mu \in L(n/p,1/(p-1)) \Longrightarrow u \in C^{0}$$ (Theorem 1)
- (C5) if $\alpha \in (0,1)$ and $p \le n$, then
  $$\begin{aligned} \mu \in {\mathcal { M}}^{\frac{n}{p-\alpha (p-1)}} \Longrightarrow |\mu |(B_\varrho )\lesssim \varrho ^{n-p+\alpha (p-1)} \Longrightarrow u \in C^{0,\alpha } \end{aligned}$$
  (estimate (64))
- (C6) if $p \le n$, then $\mu \in {\mathcal { M}}_\mathrm{b}\Longrightarrow Du \in {\mathcal {M}}^{n(p-1)/(n-1)}$ (Theorem 3)
- (C7) if $$1<q <n$$, then
  $$\begin{aligned} \mu \in L(q, \gamma ) \Longrightarrow Du \in L\left( \frac{nq(p-1)}{n-q},\gamma (p-1)\right) \end{aligned}$$
  (Theorem 3)
- (C8) if $1< q < \theta$, then $|Du|^{p-1} \in L^{\theta q/(\theta -q), \theta }$ (Theorem 3)
- (C9) $$\mu \in {\mathcal { M}}^{n} \Longrightarrow |\mu |(B_\varrho )\lesssim \varrho ^{n-1} \Longrightarrow Du \in $$ BMO (Theorem 12)
- (C10) $$\mathbf{I}_1^\mu \in L^{\infty } \Longrightarrow Du \in L^\infty $$ (Theorem 8)
- (C11) $$\mu \in L(n,1) \Longrightarrow Du \in C^{0}$$ (Theorem 3)
- (C12) if $$0< \alpha < \min \{1/(p-1), \alpha _M\}$$, then
  $$\begin{aligned} \mu \in {\mathcal { M}}^{\frac{n}{1-\alpha (p-1)}} \Longrightarrow |\mu |(B_\varrho )\lesssim \varrho ^{n-1+\alpha (p-1)} \Longrightarrow Du \in C^{0,\alpha } \end{aligned}$$
  (estimate (65)).

### *Proof*

In order to prove (C1), we begin recalling the following mapping property of the fractional maximal operator:

67 $$\begin{aligned} M_{\beta , R}:L^{t} \rightarrow L^{n t/(n-\beta t)}, \end{aligned}$$

that holds whenever $1< t < n/\beta$ (see [26, 56]). Then we go to estimate (64) and yet recall the definition of Calderón spaces given in Definition 2. This leads to determine

$$\begin{aligned} \frac{nt}{n-[p-\alpha (p-1)] t} = \frac{q}{p-1} \Longleftrightarrow t = \frac{nq}{n(p-1)+[p-\alpha (p-1)]q} \end{aligned}$$

so that $\mu \in L^t$ and (67) with the above choice implies $[M_{p-\alpha (p-1),R}(\mu )]^{1/(p-1)} \in L^{q}$. At this point (C1) follows using this fact together with estimate (64). Notice that, recalling that we are assuming $p \ge 2$, we have used that

$$\begin{aligned} t=\frac{nq}{n(p-1)+[p-\alpha (p-1)]q} > 1 \Longleftrightarrow q > \frac{n(p-1)}{n-p+\alpha (p-1)} >1. \end{aligned}$$

We have also use the fact with the previous definitions we in fact have that $t[p-\alpha (p-1)]<n$.

The assertion in (C2) follows immediately by taking $\alpha =0$ in Theorem 11 when $p=n$. We indeed recall that by the very definition of BMO spaces we have

68 $$\begin{aligned} M^{\#}_{0,R} (u)\equiv M^{\#}_R (u) \in L^\infty \Longleftrightarrow u \in \mathrm{BMO}. \end{aligned}$$

In the case $2 \le p<n$, we recall the following Hölder’s type inequality valid in Marcinkiewicz spaces ${\mathcal {M}}^t$, $t>1$:

69 $$\begin{aligned} \Vert f\Vert _{L^1(B_{\varrho })}\le \frac{t\varrho ^{n(t-1)/t}}{t-1} \Vert f\Vert _{{\mathcal {M}}^t(B_{\varrho })}\;. \end{aligned}$$

This implies that in our case $|\mu |(B_\varrho )\lesssim \varrho ^{n-p}$ holds and, by the definition of fractional maximal operator, we conclude that $M_{p,R}(\mu )$ is locally bounded. At this stage (C2) follows from estimate (44), where we again take $\alpha =0$.

The statement in (C3) is simply an obvious consequence of Theorem 1.

As for (C4), a direct computation using certain characterisation of Lorentz spaces shows that if $\mu \in L(n/p, 1/(p-1))$ for $p<n$, then (21) holds (see for instance [23] for related computations). At this stage (C4) follows by Theorem 1.

In (C5) the first implication follows again by the Hölder type inequality in (69). On the other hand, we observe that the inequality $|\mu |(B_\varrho )\lesssim \varrho ^{n-p+\alpha (p-1)}$ implies that $M_{p-\alpha (p-1),R}(\mu ) \in L^\infty$, locally, by the very definition of fractional maximal operator in Definition 6, so that (C5) follows by estimate (64).

(C6) is just an obvious consequence of the following imbedding property of Riesz potentials (see [1]):

$$\begin{aligned} \mathbf{I}_{1}:{\mathcal { M}}_\mathrm{b} \rightarrow {\mathcal { M}}^{n/(n-1)} \end{aligned}$$

and of Theorem 3.

Similarly, (C7) follows from Theorem 3 and the classical mapping property

$$\begin{aligned} \mathbf{I}_1:L(q, \gamma ) \rightarrow L(nq/(n-q),\gamma )\qquad \hbox {for}\ 1<q <n \ \hbox {and}\ \gamma >0. \end{aligned}$$

See also [26, 56].

(C8) is essentially a corollary of Theorem 3 and of the mapping property of Riesz potentials

$$\begin{aligned} \mathbf{I}_1:L^{q, \theta }\rightarrow L^{\theta q/(\theta - q),\theta }, \end{aligned}$$

originally proved by Adams [1] (see also [56] for a localization).

The first implication in (C9) is a consequence of (69) while the second follows applying Theorem 12 with $\alpha =0$. Indeed, assuming that $|\mu |(B_\varrho )\lesssim \varrho ^{n-1}$ implies that $M_1(\mu )$ is locally bounded, and therefore so is $M_{0, R}^{\#} (Du)\equiv M_{R}^{\#} (Du)$. By definition of sharp maximal function this implies that the gradient belongs to BMO, locally; see (68).

(C10) follows from Theorem 8 in an obvious way.

(C11) is again a consequence of (25) and of some basic computation involving the definition of Lorentz norm (see [22, 41]). Indeed, the condition $\mu \in L(n,1)$ allows to conclude that (25) holds, again by basic manipulations on Lorentz equivalent norms.

Finally, exactly as for the proof of (C5), (C12) follows again from (69) and estimate (65). $\square$

Some of the points in the previous corollary are well known results when considering for instance the model equation (15). The theory above allows to extend and embed them in a more general context where results follow in a unified way. Specifically, for (C2) see [55, 64], for (C6) see [7, 8, 20], for (C7) see [19, 31, 56], for (C8) see [56]. We also remark that the previous corollary is just a sample of what is possible to have using the nonlinear potential estimates approach; further spaces, as for instance Lorentz–Morrey or Besov–Morrey spaces are considerable as well (see [55, 56] for relevant definitions). More rearrangement invariant function spaces regularity results via potentials are contained in [11].

## A basic comparison estimate

This section is devoted to the proof of a comparison estimate between a considered solution $u\in W^{1,p}(\Omega )$ to (17) (notice that we are allowing for measurable coefficients here) and the function $v \in u+ W^{1,p}_0(B_R)$ defined by solving the Dirichlet problem

70 $$\begin{aligned} \left\{ \begin{array}{ll} \hbox {div}\ a(x,Dv)=0 &{} \qquad \hbox {in } \ B_{R}\\ v= u&{}\qquad \hbox {on } \partial B_{R}. \end{array}\right. \end{aligned}$$

Here $B_R \subset \Omega$ denotes a fixed ball with radius $R$. The monotonicity properties of the vector field $a(\cdot )$ described in (18) can be restated and managed more easily via the use of the auxiliary vector field $V :{\mathbb { R}}^{n} \rightarrow {\mathbb { R}}^{n}$ defined by

71 $$\begin{aligned} V(z):=(|z|^2+s^2)^{(p-2)/4}z. \end{aligned}$$

The number $s$ is the one defined in (16) and (18). Indeed, we start observing that the following inequality:

72 $$\begin{aligned} \frac{|z_1-z_2|}{c}\le \frac{|V(z_1)-V(z_2)|}{(|z_1|^2+|z_2|^2+s^2)^{(p-2)/4}} \le c|z_1-z_2| \end{aligned}$$

holds, and is valid for all matrixes $z_1,z_2 \in {\mathbb { R}}^{n}$ that are not simultaneously null (in the case $s=0$) and for every $p>1$. The constant $c$ depends only on $n$ and $p$. See [27, 55] for basic properties and for a discussion on the use of the map $V(\cdot )$ in this context. Since we are assuming $p \ge 2$, in particular we have that

73 $$\begin{aligned} |z_1-z_2|^{p/2}\le c|V(z_1)-V(z_2)|. \end{aligned}$$

Combining (18)$_2$ and (72) yields

74 $$\begin{aligned} \frac{1}{c} |V(z_1)-V(z_2)|^2 \le \langle a(x,z_2)-a(x,z_1), z_2-z_1\rangle \end{aligned}$$

again for every choice of $z_1,z_2$, $x \in \Omega$ and where $c \equiv c (n,p,\nu )$.

The lemmas in this section are already scattered in [21, 41, 55]. The proofs proposed here are anyway different and shorter. We start with a basic, weighted type energy estimate.

### **Lemma 1**

Let $u \in W^{1,p}(\Omega )$ be a solution to (17) under assumptions (18), and let $v \in u +W^{1,p}_0(B_{R})$ be as in (70). Then

75 $$\begin{aligned} \int _{B_R} \frac{|V(Du)-V(Dv)|^2}{(h + |u-v|)^{\xi }}\, d \tilde{x} \le c \frac{h^{1-\xi }}{\xi -1} |\mu |(B_R) \end{aligned}$$

holds whenever $h>0$ and $\xi >1$, where $c \equiv c (n,p,\nu )\ge 1$. The map $V(\cdot )$ has been defined in (71).

### *Proof*

The weak formulation

$$\begin{aligned} \int _{B_R} \left<a(\tilde{x},Du)-a(\tilde{x},Dv), D\varphi \right> \, d \tilde{x} = \int _{B_R} \varphi \, d\mu , \end{aligned}$$

is valid whenever $\varphi \in W^{1,p}_0(B)\cap L^{\infty }(B)$, and we use the test functions

$$\begin{aligned} \eta \equiv \eta _{\pm }:= h^{1-\xi } - (h+(u-v)_\pm )^{1-\xi }\,, \end{aligned}$$

which in fact belong to $W^{1,p}_0(B)\cap L^{\infty }(B)$. We recall the standard notation

$$\begin{aligned} (u-v)_+ := \max \{u-v,0\}\qquad \hbox {and}\qquad (u-v)_- := \max \{v-u,0\}. \end{aligned}$$

Then, notice that

$$\begin{aligned} |I_{\pm }|&:= \left| (\xi -1)\int _{B_R} \frac{\left<a(\tilde{x},Du)-a(\tilde{x},Dv),D(u-v)_\pm \right>}{(h + (u-v)_\pm )^{\xi }} \, d \tilde{x}\right| \\&= \left| ~\int _{B_R} \left<a(\tilde{x},Du)-a(\tilde{x},Dv), D\eta _{\pm } \right> \, d \tilde{x}\right| \\&= \left| ~\int _{B_R} \eta _{\pm } \, d\mu \right| \le h^{1-\xi } |\mu |(B_R) \end{aligned}$$

and therefore, using (74), we conclude with

$$\begin{aligned} (\xi -1)\int _{B_R} \frac{|V(Du)-V(Dv)|^2}{(h + |u-v|)^{\xi }}\, d \tilde{x} \le c (I_+ - I_-) \le ch^{1-\xi } |\mu |(B_R) \end{aligned}$$

so that (75) follows. $\square$

Lemma 1 and Sobolev embedding theorem in turn imply a first comparison estimate.

### **Lemma 2**

Let $u \in W^{1,p}(\Omega )$ be a solution to (17) under assumptions (18), and let $v \in u +W^{1,p}_0(B_{R})$ be as in (70). Then76and therefore77hold for constants $c_1, \tilde{c}_1 \equiv c_1,\tilde{c}_1 (n,p,\nu ,q)$, whenever

78 $$\begin{aligned} 0< q < \min \left\{ p,\frac{n(p-1)}{n-1}\right\} =:p_m. \end{aligned}$$

### *Proof*

We restrict ourselves to the proof of (76), since this obviously implies (77) via Poincaré’s inequality. Moreover, we can restrict ourselves to prove (76) assuming $q >1$, since the result for the remaining values would follow by Hölder’s inequality. Now, let us observe that we can always reduce to the situation when there exists $\xi >1$ such that

79 $$\begin{aligned} \xi q/(p-q)=q^*, \end{aligned}$$

where $q^*$ is the Sobolev conjugate of $q$. Indeed, when $p\le n$ then $p_m = n(p-1)/(n-1)$ and therefore $q< p_m$ implies that $q/(p-q)< q^* =nq/(n-q)$, so that (79) follows for the corresponding choice of $\xi$. When instead $p>n$ then we first observe that we can prove (76) in the case $n \le q < p$, since (76) for lower values of $q$ would then follow by Hölder’s inequality. Now, since by definition of Sobolev embedding exponent we can take $q^*$ as large as we please, we again find a number $\xi >1$ for which (79) holds. We then plan to apply Lemma 2 with this choice of $\xi$ and with the following one of $h$:Notice we can always assume that $h > 0$, otherwise the assertion of Lemma 2 trivializes. By using the definitions in the last two displays, inequalities (73) and (75), and finally Sobolev embedding theorem, we then haveso that (76) follows. $\square$

### *Remark 4*

By examining the arguments of the previous lemma it is possible to see that the constant $c_1$ appearing in (76) shows the following natural asymptotic for $q \rightarrow p_m$:

80 $$\begin{aligned} c_1 = c(n,p, \nu )\left( \frac{1}{p_m-q}\right) ^{q/(p-1)}, \end{aligned}$$

where $c$ is a constant that remains bounded whenever $p$ varies in a compact subset of $(1,\infty )$; see (79) and the constant appearing in Sobolev–Morrey embedding theorem. The asymptotic in (80) is typical in situations when a borderline estimate in Lebesgue spaces fails, being replaced by an estimate in Marcinkiewicz spaces; recall (58).

## A sequence of comparison estimates

Given a number $\delta _1 \in (0,1/4)$ and a ball $B(x,r)\subset \Omega$, we define the sequence of shrinking balls

81 $$\begin{aligned} B_j := B(x,r_j), \qquad \qquad r_j = \delta _1^j r, \end{aligned}$$

whenever $j \ge 0$ is an integer. The related comparison solutions $v_j \in u+W^{1,p}_0(B_j)$ are as in (70), that is

82 $$\begin{aligned} \left\{ \begin{array}{ll} \hbox {div}\ a(x,Dv_j)=0 &{} \qquad \hbox {in } \ B_{j}\\ v_j= u&{}\qquad \hbox {on } \partial B_{j}. \end{array}\right. \end{aligned}$$

Then we have

### **Lemma 3**

Let $$u \in W^{1,p}(\Omega )$$ be a solution to (17) under assumptions (18), and let $$ v_{j-1}, v_j$$ be as in (82) with $$j \ge 1$$. Suppose further that

83 $$\begin{aligned} \frac{|\mu |(B_{j-1})}{\lambda ^{p-1}r_{j-1}^{n-1}} \le 1 \end{aligned}$$

and that the bounds

84 $$\begin{aligned} \frac{\lambda }{A} \le |Dv_{j-1}| \le A \lambda \quad \hbox {in } \ B_j \end{aligned}$$

hold for constants $A\ge 1$ and $\lambda >0$. Then there exists a constant $c_2$ depending only on $n,p,\nu ,\delta _1,A$ such that85


### *Proof*

We start fixing the following quantities:

86 $$\begin{aligned} \gamma := \frac{1}{4(p-1)(n+1)} \qquad \hbox {and}\qquad \xi := 1+2\gamma , \end{aligned}$$

in view of the application of Lemmas 1 and 2, that we shall use with exponents $q$ such that

$$\begin{aligned} 0 < q \le \xi (p-1) = p-1 + \frac{1}{2(n+1)}. \end{aligned}$$

This in particular fixes the constant $c_1$ from Lemma 2. We also set

87 $$\begin{aligned} \bar{v}_{j-1} := \frac{v_{j-1}}{\lambda } \, , \qquad \bar{v}_j :=\frac{v_j}{\lambda }. \end{aligned}$$

In the rest of the proof constants denoted by $c$ will only depend on $n,p,\nu ,\delta _1,A$ and will in general vary from line to line, as usual. We start estimating the term on the left in (85) with the aid of (84) as follows:88To continue, we estimate the second-last integral appearing in the previous display. For this let us preliminary note that, by using (76) and recalling that $r_j=\delta _1 r_{j-1}$, the following estimate holds indeed for the same range of exponents $q$ working in (76):89Then, appealing to Hölder’s inequality together with (76), gives us90But now, as

91 $$\begin{aligned} \frac{(1+\gamma )(p-2)+1}{p-1} >1 \end{aligned}$$

then (83) implieswith $c \equiv c (n,p,\nu ,\delta _1)$. Combining this last estimate with (88) gives us92It remains to estimate the first term on the right hand side in the above display. Applying Hölder’s inequality, together with (72) and (75), and recalling that $\xi = 1+2 \gamma$, we obtain for any $h>0$ that93Now we choose94for some small positive $\delta \in (0,1)$ to getNotice that the presence in (94) of the parameter $\delta$ is aimed at guaranteeing that $h$ is positive; we shall eventually let $\delta \rightarrow 0$ at the end of the proof. In the above display, we use (89) and (84) to gainso that we conclude withwhere the constant $c$ ultimately depends only on $n,p,\nu ,\delta _1,A$. Plugging the last estimate in (93) now yieldswhile applying Young’s inequality and recalling again that $r_j=\delta _1 r_{j-1}$ gives95whenever $\varepsilon \in (0,1)$, where $c\equiv c(n,p,\nu ,\delta _1,A)$ is in particular independent of $\varepsilon$. It finally remains to estimate $h$, which has been defined in (94). We have96Using (76) and (89) to estimate $I_1$ leads to97In turn we bound $I_2$ appealing to (76) and using (84) repeatedly as follows:Similarly to (90) we findso thatCombining this last estimate with (97) and eventually with (96) yieldswhere $c_*$ depends only on $n,p,\nu , L, A, \delta _1$. Plugging the inequality in the above display in (95), choosing $\varepsilon = 1/(2c_*)$ and reabsorbing terms leads toNow (85) follows using this last inequality in combination with the one in display (92), and finally letting $\delta \rightarrow 0$. $\square$

## Proof of Theorems 8 and 10

The proof of Theorem 10 falls in six steps, going through Sects. 12.1–12.6 respectively. It requires the use of several different tools, and ultimately relies on a delicate iteration technique. Finally, in Sect. 12.7, we shall briefly show how to get Theorem 8 from Theorem 10.

In the following, given a ball $B \subset \Omega$, we define the excess of a vector field $f\in L^1(B,{\mathbb { R}}^k)$ over $B$ asThis functional, roughly speaking, provides an integral measure of the oscillations of the vector field $f$ in the ball $B$. An elementary property of the excess is given by the following inequality:98We remark that here and in the following we shall use the comparison estimates presented in Sects. 10 and 11 that in fact applies in particular to the case of the solutions considered in Theorem 10.

### A density property of $a$-harmonic functions

In this section we shall consider a solution $v \in W^{1,p}_\mathrm{loc}(B)\cap W^{1,1}(B)$ to the equation

99 $$\begin{aligned} \hbox {div}\, a(Dv)=0\qquad \qquad \hbox {in}\ B, \end{aligned}$$

where $B\subset {\mathbb { R}}^n$ is a given ball. We here restate in a suitable form the basic regularity properties a priori estimates for solutions to equation in (99); proofs can be for instance found in [18, 21, 51, 55]. The first is the classical gradient $L^\infty -L^1$ bound100which is valid whenever $\gamma \in (0,1)$ and for constant $c_l$ depending only on $n,p,\nu , L$. Next, we restate in suitable way the gradient Hölder continuity estimate included in display (35); more precisely, we select $\beta \equiv \beta (n,p,\nu , L) \in (0,\alpha _M)$ such that101holds whenever $\sigma B\subset B$ is a ball concentric to $B$ with $\sigma \in (0,1/2)$, such that $x_1, x_2 \in \sigma B$, where again $c_h\ge 1$ depends only on $n,p,\nu , L$. Based on the previous regularity estimates, we can prove the following result, that roughly tells that something that happens in average, happens actually everywhere, and in a precisely quantifiable way.

#### **Proposition 2**

(Density improvement) Let $v \in W^{1,p}_\mathrm{loc}(B)\cap W^{1,1}(B)$ be a solution to (99) such that102hold for some integer $k \ge 1$ and for numbers $\varGamma \ge 1$, $\lambda > 0$ and $\sigma \in (0,1/4)$ such that

103 $$\begin{aligned} 0 < \sigma \le \left( \frac{1}{4c_h\varGamma ^2}\right) ^{1/\beta }, \end{aligned}$$

where $\beta$ and $c_h$ are the constants appearing in (101). Then

$$\begin{aligned} \frac{\lambda }{4\varGamma }\le |Dv|\qquad \hbox {holds in } \ \sigma B. \end{aligned}$$

#### *Proof*

The first inequality in (102) implies that there exists a point $x_0 \in \sigma ^k B$ such that

$$\begin{aligned} |Dv(x_0)|> \frac{\lambda }{2\varGamma }. \end{aligned}$$

On the other hand, as (101) holds, the second inequality in (102) gives

$$\begin{aligned} |Dv(x)-Dv(x_0)|\le c_h \varGamma \lambda \sigma ^\beta \end{aligned}$$

whenever $x \in \sigma B$, the last two inequalities and (103) then give

$$\begin{aligned} |Dv(x)|\ge |Dv(x_0)|- |Dv(x)-Dv(x_0)|\ge \frac{\lambda }{2\varGamma } -\frac{\lambda }{4\varGamma }=\frac{\lambda }{4\varGamma } \end{aligned}$$

for all $$x\in \sigma B$$. $$\square $$

Finally, we recall two decay estimates that hold for the excess functional of the gradient and of the solutions. They are again related to the Hölder continuity of the gradient of solutions to (99) and of the solutions themselves, in the case we are considering homogeneous equations with measurable coefficients of the type

104 $$\begin{aligned} \hbox {div}\, a(x,Dv)=0\qquad \hbox {in } \ B. \end{aligned}$$

We now connect two basic regularity results for solutions to (99) and (104), respectively. They are bound to provide

#### **Theorem 17**

[19, 21, 47] Let $v \in W^{1,p}_\mathrm{loc}(B)\cap W^{1,1}(B)$ be a weak solution to (99) under the assumptions (16). There exist constants $\beta \in (0,1]$ and $c_d \ge 1$, both depending only on $n,p,\nu , L$, such that the estimate105holds whenever $\sigma B\subset B$ are concentric balls.

#### *Remark 5*

The exponent $\beta$ appearing in (105) can be chosen arbitrarily close to the exponent $\alpha _M$ appearing in (34). As a matter of fact, estimate (35) is in fact obtained as a corollary of the one in (105) via the standard Campanato’s integral characterisation of Hölder continuous functions [10]. The exponent $\alpha _M$ considered in Theorem 9 is indeed nothing but the sup of the numbers $\beta$ considerable in Theorem 17. Explicit, though not optimal, estimates on these numbers via the structure parameters $n,p,\nu ,L$, are retrievable tracking the dependence of the constants in the proofs in [19, 21, 47].

#### **Theorem 18**

Let $v \in W^{1,p}_\mathrm{loc}(B)\cap W^{1,1}(B)$ be a weak solution to (104) under the assumptions (18), and where the vector field $a:\Omega \times {\mathbb { R}}^n \rightarrow {\mathbb { R}}^n$ has measurable dependence on $x$. There exist constants $\tilde{\beta }\in (0,1]$ and $\tilde{c}_d \ge 1$, both depending only on $n,p,\nu , L$, such that the estimate106holds whenever $\sigma B\subset B$ are concentric balls, where $\tilde{r}$ is the radius of $B$.

#### *Proof*

The proof combines a few well-known facts in the regularity theory of the equation considered. Let us recall the following Caccioppoli type inequality, that states there exists a constant $c\equiv c(n,p,\nu ,L)$ such that107holds; see [40, Proposition 4.1]. On the one hand, the following gradient decay estimate holds whenever $\sigma \in (0,1/2]$ (see for instance [55, Lemma 3.3]):108where $\tilde{\beta }< \alpha _m$ and $c \equiv c (n,p,\nu , L, \tilde{\beta })$. Both the inequalities in the last displays are typical in the literature (see for instance [25, Chapter 7]) when using $L^p$-norms instead of the $L^1$-ones. Combining them then yieldsthat proves (107) in the case $\sigma \in (0,1/2]$. On the other hand, the remaining case $\sigma \in (1/2,1]$ is trivial by using (98). $\square$

#### *Remark 6*

Exactly as in Remark 5, the exponent $\tilde{\beta }$ appearing in Theorem 18 can be actually taken as close to $\alpha _m$—appearing in (30)–(31)—as we please. As a matter of fact estimates (31) and (106) are equivalent by noticing that if $v$ solves then so does $v-k$, whenever $k$ is a real number. Again, the exponent $\alpha _m$ used in Theorem 6 is actually defined as the sup of the numbers $\beta$ for which (106) works. Estimates for such numbers are available in terms on the structure parameters $n,p,\nu ,L$.

### Setting of the constants

In the following all the balls considered will be centered at the point $x \in \Omega$, and we start form a ball $B_R\equiv B(x,R)\subset \Omega$ as in the statement of the theorem. We introduce $\lambda _M$ as109and fix the constant $H\ge 1$ in a few lines (see (114) below), in a way that makes it depending only on $n,p,\nu , L$; we will then prove that

110 $$\begin{aligned} M_{1-\alpha ,R}(Du)(x)\le c\lambda _M \end{aligned}$$

holds uniformly in $\alpha \in [0,1]$, for yet another constant $c \equiv c(n,p,\nu , L)$. Clearly, we may assume without loss of generality that $\lambda _M>0$, otherwise there is nothing to prove. Let us now fix a few constants that will be relevant in the following; the constants $c_l,c_h,c_d$ have been defined in (100), (101) and Theorem 17, respectively, while $c_1$ has been introduced in Lemma 2 (that here will be used with $q=1$); all these constants depend only on $n,p,\nu , L$. Again, $\beta \equiv \beta (n,p,\nu , L) \in (0,1)$ is the exponent appearing in (101) and (105). Then we fix

111 $$\begin{aligned} \delta _1 := \left( \frac{1}{10^{8n}c_l^2c_dc_h}\right) ^{1/\beta }. \end{aligned}$$

It follows that also $\delta _1$ depends only on $n,p,\nu , L$. With such a choice of $\delta _1$ we consider the balls in  (81), that is

$$\begin{aligned} B_j := B(x,r_j), \qquad \qquad r_j = \delta _1^j r, \qquad r:=R/2, \end{aligned}$$

and the related comparison solutions $v_j \in u + W_0^{1,p}(B_j)$ in (82), that is

112 $$\begin{aligned} \left\{ \begin{array}{ll} \hbox {div}\ a(Dv_j)=0 &{} \qquad \hbox {in } \ B_{j}\\ v_j= u&{}\qquad \hbox {on } \partial B_{j}. \end{array}\right. \end{aligned}$$

Again, with $\delta _1$ now being fixed we determine the constant $c_2$ from Lemma 3 as follows:

113 $$\begin{aligned} c_2 := c_2(n,p,\nu , \delta _1, A)\quad \hbox {with } \quad A:=10^{4n}c_l. \end{aligned}$$

The following inclusions hold for every $j \ge 0$, and the will be used throughout the proof: $\delta _1B_j =B_{j+1} \subset B_j \subset (1/4)B_j \subset B_{j}.$ We finally set

114 $$\begin{aligned} H := 10^8c_1c_2c_d\delta _1^{-5n} \end{aligned}$$

and again notice that $H$ only depends on $n,p,\nu , L$. Now, let us set

$$\begin{aligned} \kappa :=\min \left\{ 1, \delta _1^{n-p+\alpha (p-1)}\right\} \qquad \hbox {and} \qquad \tilde{r}_{j}:=\frac{r_j+r_{j+1}}{2} \end{aligned}$$

for $j \ge 0$, and observe that

115 $$\begin{aligned} \mathbf{I}_{p-\alpha (p-1)}^{\mu }(x,R)&= \int _0^{2r} \frac{|\mu |(B_\varrho )}{ \varrho ^{n-p+\alpha (p-1)}} \, \frac{d\varrho }{\varrho } \nonumber \\&= \sum _{j=0}^\infty \int _{\tilde{r}_{j}}^{r_j} \frac{|\mu |(B_\varrho )}{ \varrho ^{n-p+\alpha (p-1)}} \, \frac{d\varrho }{\varrho } + \int _{8r/7}^{2r} \frac{|\mu |(B_\varrho )}{ \varrho ^{n-p+\alpha (p-1)}}\, \frac{d\varrho }{\varrho } \nonumber \\&+ \sum _{j=0}^\infty \int _{r_{j+1}}^{\tilde{r}_j} \frac{|\mu |(B_\varrho )}{ \varrho ^{n-p+\alpha (p-1)}} \, \frac{d\varrho }{\varrho } + \int _{r}^{8r/7} \frac{|\mu |(B_\varrho )}{ \varrho ^{n-p+\alpha (p-1)}}\, \frac{d\varrho }{\varrho } \nonumber \\&\ge \sum _{j=0}^\infty \int _{\tilde{r}_{j}}^{r_j} \frac{|\mu |(B_\varrho )}{ \varrho ^{n-p+\alpha (p-1)}} \, \frac{d\varrho }{\varrho } + \int _{8r/7}^{2r} \frac{|\mu |(B_\varrho )}{ \varrho ^{n-p+\alpha (p-1)}}\, \frac{d\varrho }{\varrho } \nonumber \\&\ge \kappa \sum _{j=0}^\infty \frac{|\mu |(\bar{B}_{j+1})}{r_{j+1}^{n-p+\alpha (p-1)}} \int _{\tilde{r}_{j}}^{r_j} \frac{d\varrho }{\varrho } + \frac{|\mu |(\bar{B}_{0})}{2^{n-1}r^{n-p+\alpha (p-1)}} \int _{8r/7}^{2r} \frac{d\varrho }{\varrho } \nonumber \\&\ge \delta _1^{n-1} \log \left( \frac{2}{1+\delta _1}\right) \sum _{j=0}^\infty \frac{|\mu |(\bar{B}_{j+1})}{r_{j+1}^{n-p+\alpha (p-1)}} + \frac{ \log (7/4)}{2^{n-1}}\left[ \frac{|\mu |(\bar{B}_{0})}{r_0^{n-p+\alpha (p-1)}} \right] \nonumber \\&\ge \delta _1^{n} \sum _{j=0}^\infty \frac{|\mu |(\bar{B}_{j})}{ r_{j}^{n-p+\alpha (p-1)}}. \end{aligned}$$

Therefore, by (109) and the choice in (114) it follows that

116 $$\begin{aligned} 10^8c_1c_2c_d \delta _1^{-4n}\left[ \sum _{j=0}^\infty \frac{|\mu |(\bar{B}_j)}{ r_j^{n-p+\alpha (p-1)}} \right] ^{1/(p-1)} \le \lambda _M. \end{aligned}$$

Of course we are using constants like $10^{8}$ to emphasize the fact that in certain places of the proof what it matters is to take large/small quantities. In the rest of the proof we shall denote, for every $j \ge 0$,

117 $$\begin{aligned} E_j := E(Du,B_j)\qquad \hbox {and}\qquad a_j := |(Du)_{B_{j}}| \end{aligned}$$

and

118 $$\begin{aligned} \lambda _j:= r_{j}^{\alpha -1}\lambda _M. \end{aligned}$$

### Iterating quantities

The proof is based on the fact that certain quantities, related to the fractional maximal operator we are considering, stay bounded. Specifically, we shall prove that the following inequality holds:119Let us briefly recall that the last inequality implies the one in display (110) with $c = (2/\delta _1)^{n}$. Indeed, for $0 < \varrho \le r_1$ we find $i \ge 1$ such that $r_{i+1}=\delta _1 r_i < \varrho \le r_i$, thereforeOn the other hand, if $r_1 < \varrho \le R$, recalling that $r=R/2$, then we similarly havewhere this time we have used directly (109). All in all (110) follows with $c=(2/\delta _1)^{n}$. Let us now definewhenever $j \ge 1$. Notice that, since $r_{j-1}^{1-\alpha } \le R^{1-\alpha }$ for every $j \ge 1$, then the definitions in (109) and (114) imply that120Notice that the last inequality implies, in particular, that

121 $$\begin{aligned} R^{1-\alpha }s \le \frac{\lambda _M}{100}. \end{aligned}$$

The rest of the proof is now devoted to establish (119). This will be done using induction on certain *interaction chains* defined in the next step.

### Iteration chains

We consider the set ${\mathcal {L}}$ defined by

122 $$\begin{aligned} {\mathcal {L}} := \left\{ j \in {\mathbb {N}} \ : \ C_j \le \frac{\lambda _M}{100} \right\} , \end{aligned}$$

and, accordingly, for $k\ge 1$, we then define the set

123 $$\begin{aligned} {\mathcal { C}}_i^ k= \{ j \in {\mathbb { N}}\, : i \le j \le i+k, \ i \in {\mathcal {L}}, \ i+k+1 \in {\mathcal {L}},\ \ j \not \in {\mathcal {L}} \ \hbox { if }\ j >i\}\qquad \end{aligned}$$

and call it *maximal iteration chain* of length $k$, starting at $i$. In other words, we have ${\mathcal { C}}_i^k=\{i,\ldots , i+k\}$ and each element of ${\mathcal { C}}_i^k$ but $i$ lies outside of ${\mathcal {L}}$; ${\mathcal { C}}_i^k$ is maximal in the sense that there cannot be another set of the same type properly containing it. Obviously, such sets do not exist when ${\mathcal {L}}={\mathbb { N}}$. In the same way we define

124 $$\begin{aligned} {\mathcal { C}}_i^\infty = \{ j \in {\mathbb { N}}\, : i \le j < \infty , \ i \in {\mathcal {L}}, \ j \not \in {\mathcal {L}} \ \hbox { if }\ j >i\} \end{aligned}$$

as *the infinite maximal chain* starting at $i$. Notice that, in every case, the smallest element of such a chain always belongs to ${\mathcal {L}}$, being then the only one of the chain to have such a property. Now, observe that if ${\mathcal { L}} = {\mathbb { N}}{\setminus } \{0\}$, then we are finished as in this case we have $C_j\le \lambda _M/100$ for every $j \ge 1$ and therefore125where we also used (121). Let us then assume that ${\mathcal {L}} \not = {\mathbb { N}}{\setminus } \{0\}$. Since anyway $1 \in {\mathcal { L}}$ by (120), then there must be at least one iteration chain. Let us therefore consider one of these, ${\mathcal { C}}_i^ k$, $k \le \infty$. We shall use finite induction to show that126This, due to the fact that ${\mathcal { C}}_i^k$ is arbitrary, will finally prove (119) since if a certain index $j$ does not belong to ${\mathcal { L}}$, then it must belong to some chain. On the other hand, if $j$ does not belong to any chain then it belongs to ${\mathcal { L}}$ and the corresponding inequality in (119) is automatically verified by the definition of $C_j$ and (125). We are now reduced to show the validity of (126) for any iteration chain ${\mathcal { C}}_i^k$.

### Density and decay properties along iteration chains

The idea is now that along iteration chains, a condition of the type $C_j \ge \lambda _M/100$ guarantees that the equation becomes nondegenerate and can be in a sense linearized. The consequence is that the excess functional of the gradient decays in a way that resembles the one of solutions to the Poisson equation. To implement this we need to get a few density estimates. Let us preliminary prove a *cheap decay estimate*, that actually holds also outside the context of the present proof, but whose assumptions are obviously satisfied here by (111).

#### **Lemma 4**

(Cheap decay estimate) Let the maps $v_j \in u+ W^{1,p}_0(B_j)$ be defined in (112) and the balls $\{B_j\}$ be defined in (81). Assume that the number $\delta _1$ satisfies

127 $$\begin{aligned} \delta _1 \le \left( \frac{1}{10^7c_d}\right) ^{1/\beta }. \end{aligned}$$

Then the following estimate holds for every $j \ge 0$ and for a constant depending only on $n,p,\nu , L$ but not on $\alpha \in [0,1]$:

128 $$\begin{aligned} r_{j+1}^{1-\alpha }E_{j+1} \le \frac{1}{10^6} r_{j}^{1-\alpha }E_{j} +8c_1c_d\delta _1^{-n}\left[ \frac{|\mu |(B_{j})}{ r_{j}^{n-p+\alpha (p-1)}} \right] ^{1/(p-1)}. \end{aligned}$$

Here the numbers $\{E_j\}$ have been defined in (117), and the constants $c_1, c_d$ have been introduced in (76) and (105), respectively.

#### *Proof*

By using, in order, triangle inequality, Lemma 2 and Theorem 17 with $v\equiv v_{j}$, and also using (98) repeatedly, we haveso that (4) follows by (127) and noticing that

129 $$\begin{aligned} r_{j}^{1-\alpha }\left[ \frac{|\mu |(B_{j})}{ r_{j}^{n-1}} \right] ^{1/(p-1)}=\left[ \frac{|\mu |(B_{j})}{ r_{j}^{n-p+\alpha (p-1)}} \right] ^{1/(p-1)}. \end{aligned}$$

$$\square $$

The following lemma is an easy consequence of the previous one:

#### **Lemma 5**

Let $j\ge 0$ be an integer; if

$$\begin{aligned} \delta _1^{-n}r_{j}^{1-\alpha }E_{j} \le \frac{\lambda _M}{100} \end{aligned}$$

where $\lambda _M>0$ is defined in (109), then it also holds that

130 $$\begin{aligned} \delta _1^{-n}r_{j+1}^{1-\alpha }E_{j+1} \le \frac{\lambda _M}{10^5}. \end{aligned}$$

#### *Proof*

Using (128) we have

$$\begin{aligned} \delta _1^{-n}r_{j+1}^{1-\alpha }E_{j+1}\le \frac{\delta _1^{-n}}{10^6} r_{j}^{1-\alpha }E_{j} + 8c_1c_d\delta _1^{-2n}\left[ \frac{|\mu |(B_{j})}{ r_{j}^{n-p+\alpha (p-1)}} \right] ^{1/(p-1)} \le \frac{\lambda _M}{10^5} \end{aligned}$$

where in the last estimate we have employed also (116). $\square$

The cheap decay estimate for the excess functional of Lemma 4 is not sufficient to get gradient estimates via linear Riesz potentials. Indeed, it only implies Wolff potential estimates as in (23), as shown in [21]. Anyway, by using it together with the density properties of Proposition 2 and Lemma 3, we deduce another, better decay estimate, which is this time implying linear potentials estimates.

#### **Lemma 6**

(Linearized decay estimate) Let $j\ge 1$ be an integer; if131holds and if it happens that

132 $$\begin{aligned} \delta _1^{-n}r_{j}^{1-\alpha }E_{j} \le \frac{\lambda _M}{100} \quad \hbox {and}\quad C_{j+1} \ge \frac{\lambda _M}{100} \end{aligned}$$

then we have

133 $$\begin{aligned} r_{j+1}^{1-\alpha }E_{j+1}\le \frac{1}{4} r_{j}^{1-\alpha }E_{j} + 8c_2c_d\delta _1^{-n} \lambda _M^{2-p} \left[ \frac{|\mu |(B_{j-1})}{ r_{j-1}^{n-p+\alpha (p-1)}} \right] , \end{aligned}$$

where $c_2$ depends only on $n,p,\nu , L$ and has been defined in (113).

#### *Proof*

Using triangle inequality, the second inequality in (132) and Lemma 2 we estimatewhere we have used (129) and (116) to perform the last estimations; we have also used that $r_k^{1-\alpha }\le r_{j-1}^{1-\alpha }$. The previous inequality and (130) then giveso that we conclude withand in any case, since $r_k^{1-\alpha }\le r_{j-1}^{1-\alpha }$, we have that134holds, where the numbers $\lambda _j$ have been defined in (118). On the other hand, observe that using Lemma 2 and (131) we haveand taking again (116) into account we haveApplying estimate (100) then yields

135 $$\begin{aligned} \left( |Dv_{j-1}|+s\right) \le 2^{n+1}c_l r_{j-1}^{\alpha -1}\lambda _M=2^{n+1}c_l\lambda _{j-1} \qquad \hbox {in } \ (1/2)B_{j-1}. \end{aligned}$$

We are now in position to apply the density improvement results from Proposition 2; indeed conditions in (102) are satisfied with the choices $v \equiv v_{j-1}$, $B \equiv B_{j-1}$, $\sigma = \delta _1$, $k=1$ or $k=2$ (by (134)), $\lambda \equiv \lambda _{j-1}$ and $\Gamma = 10^{3n}c_l$; observe that this is possible by the choice of $\delta _1$ made in (111). Notice that (103) is satisfied due to the choice of $\delta _1$ in (111). Proposition 2 now yields

$$\begin{aligned} \frac{\lambda _{j-1}}{10^{4n}c_l} \le |Dv_{j-1}| \qquad \hbox {in } \ B_{j}= \delta _1B_{j-1}. \end{aligned}$$

The previous inequality and (135) summarise in the following:

$$\begin{aligned} \frac{\lambda _{j-1}}{A} \le |Dv_{j-1}| \le A \lambda _{j-1} \quad \hbox {in } \ B_j\,, \qquad A=10^{4n}c_l. \end{aligned}$$

On the other hand, notice that again by (116) we have

$$\begin{aligned} \frac{|\mu |(B_{j-1})}{\lambda _{j-1}^{p-1}r_{j-1}^{n-1}} = \frac{|\mu |(B_{j-1})}{\lambda _M^{p-1}r_{j-1}^{n-p+\alpha (p-1)}} \le 1. \end{aligned}$$

We are thus in position to apply Lemma 3 thereby obtainingwhere the constant $c_2$ has been fixed in (113). With the last comparison estimate we can conclude as in Lemma 4, that is using Theorem 17 and the inequality in the last display as follows:136and the proof of (133) is complete recalling that $4c_d\delta _{1}^{\beta }\le 1/4$ by (111). $\square$

### Iteration and conclusion

Here we finally prove that (126) holds for any iteration chain ${\mathcal { C}}_i^k$, thereby concluding the proof; we remark that by construction any chain ${\mathcal { C}}_i^k$ is such that $i \ge 1$ since the numbers $C_j$ are defined only for $j \ge 1$. We shall first prove by induction that137holds for every $j \in \{i,\ldots , i+k-1\}$. Notice that by definition of iteration chains we always have $k \ge 1$. Moreover, notice also that we are going to use finite induction when $k < \infty$, that is when the length of the iteration chain is finite; when $k=\infty$ we are simply going to prove (137) for every $j \ge i$. We start by considering the case $j=i$, which is the induction basis. This follows by the very definitions of ${\mathcal { C}}_i^k$ and $C_i$ as far as (137)$_{2}$ is concerned. As for (137)$_{1}$, notice that using (121) we haveExactly in the same way we also haveThis last inequality allows to get (137)$_{3}$. Indeed, we need Lemma 6 with $j=i$; this applies by the inequality in the last display and since by definition of iteration chain ${\mathcal { C}}_i^k$ we have $C_{i+1}\ge \lambda _M/100$; note that here we are also applying (137)$_{2}$. Next, we proceed verifying the induction step; notice that this case occurs only when $k \ge 2$ while when $k=1$ the first step already concludes the proof of (137) since $\{i\}=\{i,\ldots , i+k-1\}$. We assume that (137) holds for all indexes $j \in \{i,\ldots ,h\}$ with $h< i+k-1$, and then we prove it for the index $h+1$. By Lemma 5 and (137)$_{2}$ (for $j=h$), we immediately have

138 $$\begin{aligned} \delta _1^{-n}r_{h+1}^{1-\alpha }E_{h+1} \le \frac{\lambda _M}{100}. \end{aligned}$$

Next, we are going to prove that139To this aim we start observing that, whenever $0 \le k_1<k_2$ are integers, we have140so that by letting $k_1=i$ and $k_2=h+1$ we obtain

141 $$\begin{aligned} a_{h+1} - a_{i} \le \delta _1^{-n}\sum _{j=i}^{h} E_{j}. \end{aligned}$$

By adding up inequalities (137)$_3$ for $j \in \{i,\ldots , h\}$ and yet adding $r_{i}^{1-\alpha }E_{i}$ to both sides we get

$$\begin{aligned} \sum _{j=i}^{h+1}r_{j}^{1-\alpha }E_j \le r_{i}^{1-\alpha }E_{i} + \frac{1}{4} \sum _{j=i}^{h}r_{j}^{1-\alpha }E_j + 8c_2c_d\delta _1^{-n}\lambda _M^{2-p}\sum _{j=0}^{\infty } \frac{|\mu |(B_{j})}{ r_{j}^{n-p+\alpha (p-1)}}, \end{aligned}$$

so that reabsorbing terms yields

$$\begin{aligned} \sum _{j=i}^{h+1}r_{j}^{1-\alpha }E_j \le 2r_{i}^{1-\alpha }E_{i}+ 16c_2c_d\delta _1^{-n}\lambda _M^{2-p}\sum _{j=0}^{\infty } \frac{|\mu |(B_{j})}{ r_{j}^{n-p+\alpha (p-1)}}. \end{aligned}$$

Using then (137)$_2$ for $j=i$ and (116) allows to conclude with

$$\begin{aligned} \sum _{j=i}^{h+1}r_{j}^{1-\alpha }E_j \le \frac{\lambda _M\delta _1^n}{20}. \end{aligned}$$

The last inequality together (141) gives (recall that $r_{h+1}^{1-\alpha }\le r_{j}^{1-\alpha }$ for $j\le h$)Merging the last inequality with (121) and (138), and again using Jensen’s inequality, we estimate142so that (139) follows. To complete the induction step we shall finally prove (137)$_{3}$ with $j=h+1$, that is

143 $$\begin{aligned} r_{h+2}^{1-\alpha }E_{h+2}\le \frac{1}{4} r_{h+1}^{1-\alpha }E_{h+1} + 8c_2c_d\delta _1^{-n}\lambda _M^{2-p} \left[ \frac{|\mu |(B_{h})}{ r_{h}^{n-p+\alpha (p-1)}} \right] . \end{aligned}$$

For this we want to apply Lemma 6 in the case $j=h+1$; let us verify that assumptions are satisfied. Notice that since here we are assuming that $h \le i+k-2$ then $C_{h+2} \ge \lambda _M/100$; moreover, (138) holds and by induction assumption we haveAt this point Lemma 6 applies and (143) follows. All in all, using induction (in particular, finite induction when $k< \infty$) this proves144In particular, this implies (126) when $k = \infty$, otherwise we still have to prove that145This has been on the other hand implicitly proved before. Indeed, since now (137) holds in the full range $j \in \{i,\ldots , i+k-1\}$ we repeat the computation from (141) to (142) with $h=i+k-1$, that indeed gives (145). This, together with (144), finally gives (126). The proof is complete.

### Proposition 1 and Theorem 8

#### *Proof of Proposition 1*

Let us define $\varrho _i=r/2^i$, for every integer $i \ge 0$ and $r=101|x-y|/100$. Then observe that146and, upon summation, we get

147 $$\begin{aligned} \sum _{i\ge 0} |(f)_{B(x, \varrho _i)}-(f)_{B(x, \varrho _{i+1})}|&\le c(n) M^{\#}_{\alpha ,R}(f)(x)\sum _{i\ge 0} \varrho _i^\alpha \nonumber \\&\le \frac{c}{\alpha }M^{\#}_{\alpha ,R}(f)(x)|x-y|^\alpha . \end{aligned}$$

We have used the elementary inequality

$$\begin{aligned} \sum _{i\ge 0} \frac{1}{2^{i\alpha }}\le \frac{c}{\alpha }. \end{aligned}$$

Notice that this shows that $\{(f)_{B(x, \varrho _i)}\}$ is a Cauchy sequence and therefore the following limit exists:

148 $$\begin{aligned} \lim _{i \rightarrow \infty } (f)_{B(x,\varrho _i)}. \end{aligned}$$

Let us now show that the limit

149 $$\begin{aligned} \lim _{\varrho \rightarrow 0} (f)_{B(x,\varrho )} \end{aligned}$$

exists too, and therefore defines the precise representative of $f$ and $x$, which is indeed denoted by $f(x)$. To this aim, take $0 <\varrho \le \varrho _0$. There exists an integer $i$ such that $\varrho _{i+1}< \varrho \le \varrho _i$; therefore, as for (146), we have

$$\begin{aligned} |(f)_{B(x, \varrho _i)}-(f)_{B(x, \varrho )}| \le c(n) M^{\#}_{\alpha ,R}(f)(x) \varrho ^\alpha . \end{aligned}$$

The last inequality, together with the existence of the limit in (148), proves the existence of the limit in (149). Changing in the previous argument $x$ by $y$ and choosing this time $r=|x-y|/100$ gives

150 $$\begin{aligned} \sum _{i\ge 0} |(f)_{B(y, \varrho _i)}-(f)_{B(y, \varrho _{i+1})}| \le \frac{c}{\alpha } M^{\#}_{\alpha ,R}(f)(y)|x-y|^{\alpha } \end{aligned}$$

and in the same way we can prove that the precise representative of $f$ can be defined at $y$. Notice now that telescoping summation and (147) gives

$$\begin{aligned} |f(x)-(f)_{B(x, \varrho _{0})}|\le \sum _{i\ge 0} |(f)_{B(x, \varrho _i)}-(f)_{B(x, \varrho _{i+1})}| \le \frac{c}{\alpha } M^{\#}_{\alpha ,R}(f)(x)|x-y|^{\alpha } \end{aligned}$$

with a similar estimate for balls centred at $y$ following by (150). Therefore, using triangle inequality, and recalling the choices of $r$ made for the points $x$ and $y$, we conclude with

$$\begin{aligned} |f(x)-f(y)|&\le |(f)_{B(y, 101|x-y|/100)}-(f)_{B(y, |x-y|/100)}|\\&+ \frac{c}{\alpha }\left[ M^{\#}_{\alpha ,R}(f)(x)+M^{\#}_{\alpha ,R}(f)(y)\right] |x-y|^{\alpha }. \end{aligned}$$

To estimate the first term in the right hand side of the previous inequality we proceed as follows:Collecting the inequalities in the last two displays yields (43). Notice that in the last display we have used that $B(y, |x-y|/100)\subset B(x, 101|x-y|/100)$. We have also used that

$$\begin{aligned} \frac{101|x-y|}{100}\le \frac{404R}{500} \le R \qquad \hbox {and} \qquad B(x, 101|x-y|/100) \subset B_{8R/5}. \end{aligned}$$

#### *Proof of Theorem 8*

Applying Proposition 1 in the ball $B_{R/4}$ and then (42), we get that the estimate151holds for every choice $x, y \in B_{R/4}$. The constant $c$ appearing in the previous estimate just depends on $n$, but is independent of $\alpha \in [0,1]$. In order to estimate the last two integrals, we recall the following Caccioppoli type inequality:152that holds whenever $\gamma B \subset \Omega$ is a ball with radius $\gamma r$, and $\gamma >1$. The constant $c$ depends only on $n,p,\nu , L$ and $\gamma$. See [40, Proposition 4.1] for a proof. We apply (152) to $B(x,5R/8)$ and $B(y,5R/8)$ with the choice $\gamma = 7/6$ and we use it in (151). Observe that this is possible since

$$\begin{aligned} x, y \in B_{R/4} \Longrightarrow \gamma B(x,5R/8) \cup \gamma B(y,5R/8) \subset B_R. \end{aligned}$$

Then we notice that the following elementary estimate holds whenever $\sigma \in (0,1)$:

$$\begin{aligned}&R\left[ \frac{|\mu |(B(x, \sigma R))}{(\sigma R)^{n-1}}\right] ^{1/(p-1)}\left( \frac{|x-y|}{R}\right) ^\alpha \\&\qquad \le -\frac{1}{\min \{\sigma ^{n-1}, \sigma ^{(p-1)(1-\alpha )}\}\log \sigma }\left[ \,\,\int _{\sigma R}^R\frac{|\mu |(B(x, \varrho ))}{\varrho ^{n-p+\alpha (p-1)}}\, \frac{d\varrho }{\varrho }\right] ^{1/(p-1)}|x-y|^\alpha \\&\qquad \le c(\sigma )\left[ \mathbf{I}_{p-\alpha (p-1)}^\mu (x,R)\right] ^{1/(p-1)}|x-y|^{\alpha } \end{aligned}$$

(with a similar one centred at $y$), and, using it with $\sigma = 35/48$, we finally conclude with (33).$\square$

#### *Remark 7*

There is a variant of estimate (33), which is now uniform in the whole range $\alpha \in [0,1]$, and it is the following:153The constant $c$ appearing in the above estimate is this time independent of $\alpha$, and only exhibits a dependence on $n,p,\nu , L$. Estimate (153) follows exactly as estimate (33), but tracking the dependence on the constants on $\alpha$ starting from (151). Estimate (153) describes in a precise way the blow-up rate of the constant $c$ in (33) when $\alpha \rightarrow 0$. More precisely, the coefficient $1/\alpha$ appears to be related to the loss of $L^\infty$-estimates towards BMO-ones already discussed after Proposition 1. Moreover, it is the same blow-up rate appearing when fractional estimates get lost as the fractional differentiability parameter disappears. See for instance [52].

## Proof of Theorem 12

This theorem has been proved in [40] in a slightly different form and with a different proof; Theorem 12 is essential for the proof of Theorem 9. We consider the sequence of shrinking balls introduced in (81) with the corresponding solutions $v_j \in u+ W_0^{1,p}(B_i)$ defined in (112); the number $\delta _1 \in (0, 1/4)$ is this time chosen as

154 $$\begin{aligned} \delta _1 := \left( \frac{\varepsilon }{4c_d}\right) ^{1/(\beta -\tilde{\alpha })},\qquad \beta := \frac{\alpha _M+\tilde{\alpha }}{2}\,, \end{aligned}$$

while we take $r:=R$. Here we take $\varepsilon \in (0,1/2)$ to be a fixed number. Finally, we recall the definition of the excess quantities $\{E_j\}$ in (117). Before going on let us record an identity of later use, that is

155 $$\begin{aligned} r_j^{-\alpha }\left[ \frac{|\mu |(B_{j})}{r_j^{n-1}}\right] ^{1/(p-1)}= \left[ \frac{|\mu |(B_{j})}{r_j^{n-1+\alpha (p-1)}}\right] ^{1/(p-1)}. \end{aligned}$$

We are now going to use estimate (105) as we have done for Lemma 4. In particular, in light of Remark 5, with $\tilde{\alpha }< \alpha _M$, we can choose in (105) a number $\beta$ such that $\tilde{\alpha }< \beta < \alpha _M$ and we take the one in (154). Therefore, recalling that $r_{j+1}^{-\alpha }=\delta _1^ {-\alpha }r_{j}^{-\alpha }$, we haveBy recalling (154) we conclude with

156 $$\begin{aligned} r_{j+1}^{-\alpha }E_{j+1} \le \varepsilon r_{j}^{-\alpha }E_{j}+c\left[ \frac{|\mu |(B_{j})}{r_j^{n-1+\alpha (p-1)}}\right] ^{1/(p-1)} \end{aligned}$$

where $c \equiv c(n,p,\nu , L, \tilde{\alpha })$ as a consequence of the fact that the choice in (154) determines a number $\delta _1$ which again depends on $n,p,\nu , L, \tilde{\alpha }$ and $\varepsilon$ via the dependence of the quantities $\beta$ and $c_d$. Observe that the resulting estimate is independent of $\alpha$ as long as this belongs to $[0,\tilde{\alpha }]$. From (156) it follows that

157 $$\begin{aligned} \sup _{j \ge 1} \, r_{j}^{-\alpha }E_{j} \le \varepsilon \sup _{j \ge 1} \, r_{j}^{-\alpha }E_{j} +\varepsilon R^{-\alpha }E_{0}+c(\varepsilon )\sup _{j \ge 0}\,\left[ \frac{|\mu |(B_{j})}{r_j^{n-1+\alpha (p-1)}}\right] ^{1/(p-1)}.\nonumber \\ \end{aligned}$$

Taking for instance $\varepsilon = 1/4$, reabsorbing terms and yet adding $R^{-\alpha }E_{0}$ to both sides of the resulting inequality, we conclude with158This last estimate implies the statement of the theorem, that is

159 $$\begin{aligned} M_{\alpha , R}^{\#} (Du)(x) \le cS\,, \end{aligned}$$

where $c\equiv c (n,p,\nu , L, \tilde{\alpha })$ and $S$ in fact denotes the quantity appearing in the right hand side of (158). Indeed, let us consider a positive radius $\varrho \le R$, and determine $j \ge 0$ such that $r_{j+1}< \varrho \le r_j$; then using also (98) we haveand (159) follows keeping in mind the dependence of $\delta _1$ on $n,p,\nu , L,\tilde{\alpha }$. The proof is complete.

### A VMO-type result

A modification to the proof of Theorem 12 allows to get a corollary that is interesting in itself and that will be useful in order to obtain subsequent results.

#### **Theorem 19**

(VMO gradient regularity) Let $u \in W^{1,p}(\Omega )$ be a weak solution to the Eq. (13) under the assumptions (16). Assume that160and

161 $$\begin{aligned} \lim _{R\rightarrow 0}\frac{|\mu |(B(x,R))}{R^{n-1}}=0 \end{aligned}$$

hold for a certain $x \in \Omega$, then162Moreover, if $Du$ is locally bounded and if the limit in (161) is uniform with respect to $x$ then $Du$ is (locally) VMO in $\Omega$.

#### *Proof*

In order to prove (162) we show that, for every choice of $\tilde{\varepsilon }$, there exists $\tilde{R}>0$, depending on $n,p,\nu , L, \tilde{\varepsilon }, \mu$ and the point $x$, such that

163 $$\begin{aligned} \varrho \le \tilde{R} \Longrightarrow E(Du, B(x,\varrho ))< \tilde{\varepsilon }. \end{aligned}$$

We revisit the proof of Theorem 12, where we consider the case $\alpha =\tilde{\alpha }= 0$. Assume that $B(x,R) \subset \Omega$. Estimate (157) then gives

$$\begin{aligned} \sup _{j \ge 1} \,E_{j} \le 2\varepsilon E(Du, B(x,R))+c(\varepsilon )\left[ M_{1,R}(\mu )(x)\right] ^{1/(p-1)}, \end{aligned}$$

which is valid as soon as $\delta _1$ and $\varepsilon$ are linked as in (154), and $\varepsilon \in (0,1/2)$. In particular, we also have

164 $$\begin{aligned} \sup _{j \ge 1} \,E_{j} \le 4\varepsilon S_1+c(\varepsilon )\left[ M_{1,R}(\mu )(x)\right] ^{1/(p-1)}, \end{aligned}$$

where we have used the abbreviation $S_1 \equiv S_1(x)$. Now, let us notice how the previous argument implies that

165 $$\begin{aligned} \sup _{\varrho \le \delta _1R} \,E(Du, B(x,\varrho )) \le 4\varepsilon S_1+c(\varepsilon )\left[ M_{1,R}(\mu )(x)\right] ^{1/(p-1)} \end{aligned}$$

holds for every $\varrho \le \delta _1R=r_1$ and $\delta _1$ is again chosen as in (154). Indeed, consider a number $\varrho \le \delta _1R$ with $\tilde{\alpha }=0$; then there exists an integer $k\ge 1$ such that $r_{k+1}< \varrho \le r_{k}$. This means that $\varrho = \delta _1^kR'$ for some $R' \in (\delta _1R, R]$. Now, in the proof of Theorem 12 replace $R$ by $R'$ and we gain a new chain of cylinders $B_{j}$ for which (164) obviously holds; indeed, notice that $M_{1,R'}(\mu )(x) \le M_{1,R}(\mu )(x)$ since $R' \le R$. With this new definition we have $E(Du, B_{\varrho })= E_k$ and therefore (165) is nothing but (164) with this new choice of the radii $\{r_j\}\equiv \{\delta _1^jR'\}$. All in all we have proved that (165) holds with $\delta _1$ depending only on $n,p,\nu , L$ and $\varepsilon$. Let us recall that estimate (165) holds with any choice of the initial radius $R$, which is a free parameter that is going to be chosen in the next lines; notice indeed that all the constants determined up top now are independent of the starting radius $R$. Now, first choose $\varepsilon \equiv \varepsilon (n,p,\nu , L, \tilde{\varepsilon })$ small enough in order to have

166 $$\begin{aligned} 4\varepsilon S_1 \le \frac{\tilde{\varepsilon }}{2}. \end{aligned}$$

This determines the constant $c(\varepsilon )$ as a function of $n,p,\nu , L, \tilde{\varepsilon }$; notice that this is possible by the first assumption in (161), while using the second we can determine a radius $R_1$, depending essentially on $n,p,\nu , L,\tilde{\varepsilon }$ and the point $x$ (via the rate of convergence in (161)), such that

167 $$\begin{aligned} R \le R_1 \Longrightarrow c(\varepsilon )\left[ M_{1,R}(\mu )(x)\right] ^{1/(p-1)}\le \frac{\tilde{\varepsilon }}{2}. \end{aligned}$$

Combining the content of the last two displays with the one of (165) (where we now take with $R \equiv R_1$), and recalling the definition of the excess functional $E(Du, B_R)$, yields (163) with the choice $\tilde{R} = \delta _1R_1$. As for the local VMO-regularity of $Du$ we recall that this means that, for every $\tilde{\varepsilon }$, the choice of $\tilde{R}$ in (163) can be done independently of the point $x$ as long as $B_{\tilde{R}}$ varies in a fixed compact subset $\Omega ' \Subset \Omega$. We can now check this by showing that all the choices of the constants above can be done independently of the point $x$, but depending only on the fixed subset $\Omega '$. Indeed (166) can be replaced by

168 $$\begin{aligned} 4\varepsilon S_1\le 4\varepsilon \Vert Du\Vert _{L^{\infty }(\Omega ')} \le \frac{\tilde{\varepsilon }}{2}. \end{aligned}$$

The choice of $R_1$ in (167) can be made uniform as well by  (161), which is now uniform with respect to $x$, and the proof of the VMO-regularity of $Du$ is complete, too.

## Proof of Theorem 15

We first prove the theorem in the case we have an energy solution $u \in W^{1,p}(\Omega )$; this will be done through Steps 14.1–14.3. Then, in the final Step 14.4, we describe the approximation argument to prove the theorem for any SOLA $u \in W^{1,p-1}_0(\Omega )$ to problem (55). We indeed prefer a separate treatment of the two cases since in this second part we shall also present the general arguments to prove that the other potential estimates presented in this paper for energy solutions actually hold for SOLA too.

### Proof in the case of energy solutions: beginning

We fix a ball $B_R \equiv B(x,R)\subset \Omega$ and then consider in the rest of the proof balls which are concentric to it. We notice that by assumption (60) the quantity169is finite, where $c \equiv c(n,p,\nu , L)$ is the constant appearing in (40). We shall then prove that, for every $\varepsilon >0$, there exists a radius $r_\varepsilon \le R$, in general depending only on $n,p,\nu , L$ and the point $x$, such that

170 $$\begin{aligned} |(Du)_{B(x,\varrho )}-(Du)_{B(x,\tau )}|\le \lambda _M\varepsilon \qquad \hbox {holds whenever } \ 0< \tau < \varrho \le r_\varepsilon . \end{aligned}$$

This will finally prove (61). Notice that with the above definitions, by (40) we have171while, and this time by Theorem 19, we have that

172 $$\begin{aligned} \lim _{\varrho \rightarrow 0}\,E(Du, B(x,\varrho ))=0. \end{aligned}$$

Notice that, in order to verify the assumptions in (161) we have implicitly used the fact that $\mathbf{I}_1^\mu (x,R)< \infty$ implies that

173 $$\begin{aligned} \lim _{\varrho \rightarrow 0} \, \mathbf{I}_1^\mu (x,\varrho )=0 \end{aligned}$$

and this, thanks to an argument similar to the one on (115), also implies

174 $$\begin{aligned} \lim _{\varrho \rightarrow 0} \, \frac{|\mu |(B(x,\varrho ))}{\varrho ^{n-1}}=0 \end{aligned}$$

which is in fact the second part of the main assumption in Theorem 161.

### Setting of the constants and a sequence

We revisit the arguments of the proof of Theorem 10, while in the following the number $\varepsilon$ is fixed as the one introduced in (170). As usual the constants $c_l,c_h,c_d$ have been defined in (100), (101) and (105), respectively; $c_1$ is from Lemma 2 (where we take $q=1$) and $\beta \equiv \beta (n,p,\nu , L) \in (0,1)$ appears in (101) and (105). We this time fix $\delta _1 \equiv \delta _1(n,p,\nu , L, \varepsilon )$ as

175 $$\begin{aligned} \delta _1 := \left( \frac{\varepsilon ^2}{10^{8n}c_l^2c_dc_h}\right) ^{1/\beta }. \end{aligned}$$

With such a choice of $\delta _1$ we determine the constant $c_2$ from Lemma 3 as follows:

176 $$\begin{aligned} c_2 := c_2(n,p,\nu , L, \delta _1, A)\qquad \hbox {with} \qquad A:=\frac{10^{4n}c_l}{\varepsilon }. \end{aligned}$$

Notice that all the constants appearing up to now show a global dependence on the constants $n,p,\nu , L$ and $\varepsilon$. Now we can take a radius $R_l\equiv R_l(n,p,\nu , L, \varepsilon , x)\le R$ such that the following two conditions are satisfied:

177 $$\begin{aligned} \left[ \int _0^{2 R_l} \frac{|\mu |(B(x,\varrho ))}{\varrho ^{n-1}} \, \frac{d\varrho }{\varrho } \right] ^{1/(p-1)}\le \frac{\delta _1^{5n} \lambda _M\varepsilon }{10^8 c_1c_2c_d} \end{aligned}$$

and

178 $$\begin{aligned} \sup _{0<\varrho \le R_l} \, E(Du, B(x,\varrho )) \le \frac{ \delta _1^{5n}\lambda _M\varepsilon }{10^8}. \end{aligned}$$

Notice that we can satisfy (177) by (173), while the choice in (178) is possible due to (172). Finally, for integers $j \ge 0$ we consider the balls as in  (81) where this time

$$\begin{aligned} B_j := B(x,r_j)\,, \quad r_j = \delta _1^jr \,, \quad r:=R_l \end{aligned}$$

and the related comparison solutions $v_j \in u + W_0^{1,p}(B_j)$ in (112). Accordingly, the numbers $\{E_i\}$ are again defined as in (117). Notice also that (177) and a computation totally similar to the one in (115) (where we take $\alpha =1$) give

179 $$\begin{aligned} \left[ \sum _{j=0}^\infty \frac{|\mu |(\bar{B}_j)}{ r_j^{n-1}} \right] ^{1/(p-1)}\le \frac{\delta _1^{4n} \lambda _M\varepsilon }{10^8 c_1c_2c_d}. \end{aligned}$$

In Step 14.3 below, we will prove that

180 $$\begin{aligned} |(Du)_{B_h}-(Du)_{B_k}|\le \frac{\lambda _M\varepsilon }{12}\qquad \hbox {holds whenever } \ 2 \le k \le h. \end{aligned}$$

Let us assume for a moment that (180) and let us finish the proof of (170) with the choice

181 $$\begin{aligned} r_\varepsilon :=\delta _1^2 R_l. \end{aligned}$$

Indeed, let us fix $0 < \tau < \varrho \le r_\varepsilon$. This means that there exist two integers $k$ and $h$, such that $2 \le k \le h$,

182 $$\begin{aligned} \delta _1^{k+1}R_l < \varrho \le \delta _1^{k}R_l\qquad \hbox {and} \qquad \delta _1^{h+1}R_l < \tau \le \delta _1^{h}R_l \end{aligned}$$

hold. Applying (178), and taking (182) into account, we getSimilarly, we also gain

$$\begin{aligned} |(Du)_{B(x, \tau )}- (Du)_{B_{h+1}}|\le \frac{\lambda _M\varepsilon }{10}. \end{aligned}$$

Using the inequalities in the last two displays together with (180) and triangle inequality establishes (170). It remains to prove (180) and this will be done in the next step.

### Non-degenerate iteration chains and proof of (180)

We set, for $j \ge 1$
and then, as in (122), we define

183 $$\begin{aligned} {\mathcal {L}} := \left\{ j \in {\mathbb {N}} \ : \ C_j \le \frac{\lambda _M\varepsilon }{100} \right\} . \end{aligned}$$

Accordingly, we also define the chains ${\mathcal { C}}_i^ k$ and ${\mathcal { C}}_i^ \infty$ as in (123) and (124), respectively. The difference here is the appearance of the parameter $\varepsilon$ in the new definition of the set ${\mathcal { L}}$ in (183); this allows to interpret the sets ${\mathcal { C}}_i^ k$ as maximal chains along which the equation becomes nondegenerate. In this case it will be useful to consider the following number:

184 $$\begin{aligned} j_m := \min \, {\mathcal {L}}. \end{aligned}$$

Needless to say, in case ${\mathcal {L}}$ is empty we have $j_m = \infty$ and this is actually a favourable case since the problem never becomes degenerate. The following lemma is similar to Lemma 6:

#### **Lemma 7**

Let $j\ge 1$ be an integer such that

$$\begin{aligned} C_{j+1} \ge \frac{\lambda _M\varepsilon }{100} \end{aligned}$$

holds; then we have

185 $$\begin{aligned} E_{j+1}\le \frac{\varepsilon }{4} E_{j} + 8c_2c_d\delta _1^{-n} \lambda _M^{2-p} \left[ \frac{|\mu |(B_{j-1})}{ r_{j-1}^{n-1}} \right] \,, \end{aligned}$$

where $c_2$ depends only on $n,p,\nu , L, \varepsilon$ and has been defined in (176).

#### *Proof*

As for Lemma 6 we estimate186where we used (76) and (179). On the other hand, using triangle inequality, (171), (179) and yet Lemma 2, we haveApplying estimate (100) with $v \equiv v_{j-1}$ then yields

187 $$\begin{aligned} \left( |Dv_{j-1}|+s\right) \le 2^{n+1}c_l \lambda _M \qquad \hbox {in } \ B_{j-1}/2. \end{aligned}$$

In view of (186)–(187) we are in position to apply Proposition 2 with the choices $v \equiv v_{j-1}, B \equiv B_{j-1}, \sigma = \delta _1, k=1, \lambda \equiv \lambda _M$ and $\varGamma = 10^{3n}c_l /\varepsilon$; observe that this is again possible by the choice of $\delta _1$ made in (175). This yields

$$\begin{aligned} \frac{\lambda _{M}\varepsilon }{10^{4n}c_l} \le |Dv_{j-1}| \qquad \hbox {in } \ B_{j} \end{aligned}$$

The inequality in the above display and (187) summarise in the following line:

$$\begin{aligned} \frac{\lambda }{A} \le |Dv_{j-1}| \le A \lambda \quad \hbox {in } \ B_j, \qquad A=\frac{10^{4n}c_l}{\varepsilon } \end{aligned}$$

and therefore we are in position to apply Lemma 3 thereby obtainingwhere the constant $c_2$ has been fixed in (176). With the last comparison estimate we can conclude with (185) as in (136). $\square$

We now proceed with the proof of (180), obviously assuming $k < h$. We then analyse three different cases.

*Case 1:* $k < h \le j_m$, where we recall that the number $j_m$ has been defined in (184). By the very definition of $j_m$ it follows that $C_{j+1}\ge \lambda _M\varepsilon /100$ holds for every $j \in \{k-1, \ldots , h-2\}$ and therefore we apply Lemma 7 to get that

188 $$\begin{aligned} E_{j+1} \le \frac{1}{4} E_j + 8c_2c_d\delta _1^{-n} \lambda _M^{2-p} \left[ \frac{|\mu |(B_{j-1})}{ r_{j-1}^{n-1}} \right] \end{aligned}$$

holds, again for every $j \in \{k-1, \ldots , h-2\}$. Summing up yields

$$\begin{aligned} \sum _{j=k}^{h-1} E_j \le \frac{1}{4}\sum _{j=k-1}^{h-2} E_j +8c_2c_d\delta _1^{-n} \lambda _M^{2-p} \sum _{j=0}^{\infty } \frac{|\mu |(B_{j})}{ r_{j}^{n-1}} \end{aligned}$$

so that

$$\begin{aligned} \sum _{j=k}^{h-1} E_j \le \frac{1}{3} E_{k-1} +16c_2c_d\delta _1^{-n} \lambda _M^{2-p} \sum _{j=0}^{\infty } \frac{|\mu |(B_{j})}{ r_{j}^{n-1}}. \end{aligned}$$

Finally, recalling (178) and (179), we conclude with

$$\begin{aligned} \sum _{j=k}^{h-1} E_j \le \frac{\delta _1^{2n}\lambda _M\varepsilon }{50}. \end{aligned}$$

On the other hand, taking $k_1= k$ and $k_2=h$ in (140) and using the inequality in the last display we can estimate

189 $$\begin{aligned} |(Du)_{B_h}-(Du)_{B_k}|\le \delta _1^{-n}\sum _{j=k}^{h-1} E_{j}\le \lambda _M\varepsilon \end{aligned}$$

and (180) follows.

*Case 2:* $j_m\le k < h$. Here (180) follows as a consequence of the inequalities

190 $$\begin{aligned} |(Du)_{B_h}| \le \frac{\lambda _M\varepsilon }{25}\qquad \hbox {and}\qquad |(Du)_{B_k}|\le \frac{\lambda _M\varepsilon }{25}. \end{aligned}$$

If $h \in {\mathcal {L}}$, the first inequality in (190) follows immediately from the definition of ${\mathcal {L}}$; we can therefore assume $h \not \in {\mathcal {L}}$. Then, as $h > j_m$, it is possible to consider a nondegenerate iteration chain ${\mathcal { C}}_{i_h}^{m_h}$ with $m_h>0$, such that $h \in {\mathcal {C}}_{i_h}^{m_h}$; notice that $h > i_h$ as $h \not \in {\mathcal {L}} \ni i_h$, since by definition the first index in a chain does belong to ${\mathcal { L}}$. We are again in position to apply Lemma 7 for $j \in \{i_h, \ldots , i_h+m_h-1\}$, so that (188) holds for the corresponding indexes. Summing up, proceeding as after (188), and yet summing $E_{i_h}$ to both sides of the resulting inequality, we arrive at

$$\begin{aligned} \sum _{j=i_h}^{i_h+m_h} E_j \le 2E_{i_h} +8c_2c_d\delta _1^{-n} \lambda _M^{2-p} \sum _{j=0}^{\infty }\frac{|\mu |(B_{j})}{ r_{j}^{n-1}} \le \frac{\delta _1^{2n}\lambda _M\varepsilon }{50}. \end{aligned}$$

We have again used (178) and (179). Proceeding as in (140) (taking $k_1= i_h$ and $k_2=h$), and using the definition of ${\mathcal { C}}_{i_h}^{m_h}$ to estimate

$$\begin{aligned} |(Du)_{B_{i_h}}|\le C_{i_h}\le \frac{\lambda _M\varepsilon }{100} \end{aligned}$$

we have

$$\begin{aligned} |(Du)_{B_h}|&\le |(Du)_{B_h}-(Du)_{B_{i_h}}| +|(Du)_{B_{i_h}}|\nonumber \\&\le \delta _1^{-n} \sum _{i=i_h}^{h-1} E_i + \frac{\lambda _M\varepsilon }{100}\nonumber \\&\le \delta _1^{-n} \sum _{i=i_h}^{i_h+m_h} E_i+ \frac{\lambda _M\varepsilon }{100} \le \frac{\lambda _M\varepsilon }{25}, \end{aligned}$$

that is the first inequality in (190). The proof of the second inequality in (190) is completely similar; we just observe that we can assume that $k >j_m$ otherwise the inequality itself is trivial as $k \in {\mathcal { L}}$.

*Case 3:* $k < j_m < h$. This can be actually treated by a combination of the first two cases. It is indeed sufficient to prove that the inequalities in display (190) still hold. The first inequality in (190) follows exactly as in Case 2. As for the second estimate in (190), let us remark that, as $j_m \in {\mathcal {L}}$, we have that

$$\begin{aligned} |(Du)_{B_{j_m}}| \le C_{j_m}\le \frac{\lambda _M\varepsilon }{50}. \end{aligned}$$

On the other hand, we can repeat the argument of Case 1, with $h$ replaced by $j_m$, iterating from $h$ to $j_m$ and thereby obtaining

$$\begin{aligned} |(Du)_{B_{j_m}}-(Du)_{B_k}| \le \frac{\lambda _M\varepsilon }{50} \end{aligned}$$

as in (189). Finally, the inequalities in the last two displays and yet triangle inequality give

$$\begin{aligned} |(Du)_{B_k}| \le |(Du)_{B_k}-(Du)_{B_{j_m}}|+|(Du)_{B_{j_m}}| \le \frac{\lambda _M\varepsilon }{50}+\frac{\lambda _M\varepsilon }{100} \le \frac{\lambda _M\varepsilon }{25} \end{aligned}$$

and therefore the second inequality in (190) follows. The proof of Theorem 15 is therefore complete in the case of an energy solution $u \in W^{1,p}(\Omega )$.

### Very weak solutions

In this section we are going to prove Theorem 15 for SOLA. We shall also obtain the extensions of Theorems 10 and 19 to SOLA, since these are indeed needed as preliminary results. The method we propose here is of course based on an approximation argument, but, rather than passing to the limits in the final estimates, we will pass to the limits “in the proofs”. Strictly speaking, we shall pass to the limits in some estimates from the proofs. The same methods developed for energy solutions will then work for SOLA. We start with a preliminary result. Let us consider a ball $B(x,R) \subset \Omega$, and a sequence of shrinking balls $\{B_j\}$, concentric to $B(x,R)$:

191 $$\begin{aligned} \cdot \cdot \cdot B_{j+1} \subset B_{j} \subset B_{j-1}\cdot \cdot \cdot B_0 \subset B(x,R). \end{aligned}$$

Then we have the following:

#### **Lemma 8**

Let $u$ be a SOLA to (17) under the assumptions (18), and let $\{B_j\}$ be the sequence of balls considered in (191). There exists a sequence of functions $\{v_j\}$ such that for every $j \ge 0$ it holds

- $$ v_j \in W^{1,p}_\mathrm{loc}(B_j) \cap u+ W^{1,p-1}_0(B_j) $$
- $$ v_j \ \hbox { is a local energy solution in } B_j \hbox { i.e. }\ \hbox {div}\, a(x,Dv_j)=0\ \ \hbox {in} B_j$$
- There exist constants $c_1, \bar{c}_1\equiv c_1, \bar{c}_1(n,p,\nu )$ such that 192 and 193 hold whenever $q$ is as in (78)
- There exists a constant $c \equiv c(n,p,\nu )$ such that
  194 $$\begin{aligned} \int _{B_j} \frac{|V(Du)-V(Dv_j)|^2}{(h + |u-v_j|)^{\xi }}\, d \tilde{x} \le c \frac{h^{1-\xi }}{\xi -1} |\mu |(\bar{B}_j) \end{aligned}$$
  holds whenever $h>0$ and $\xi >1$, where $c \equiv c (n,p,\nu )\ge 1$. The function $V(\cdot )$ has been defined in (71).

#### *Proof*

The proof goes via approximation and we confine ourselves to explain the arguments for the inequality in display (192), those for the other ones being completely analogous. We preliminary recall that by the results in [8, 55]—see also Theorem 14—any SOLA $u$ to (55) belongs to $W^{1,q}_\mathrm{loc}(\Omega )$ for the numbers $q$ in the range described in (76). With $u$ being now a fixed SOLA to (17), we consider the associated sequence $\{u_k\} \subset W^{1,p}_\mathrm{loc}(\Omega )$ from Definition 8 and note that using a standard diagonal we may assume, up to passing to not relabelled subsequences, that $Du_k \rightarrow Du$ a.e.. With $B_j$ being fixed we now build the function $v_j$ as required in the statement of the lemma. We start defining $w_k \in u_k +W^{1,p}_0(B_j)$ as the unique solution to the Dirichlet problem

195 $$\begin{aligned} \left\{ \begin{array}{ll} \hbox {div}\ a(x,Dw_k)=0 &{} \qquad \hbox {in } B_j\\ w_k= u_k&{}\qquad \hbox {on } \partial B_j. \end{array}\right. \end{aligned}$$

Applying Lemma 2 in this context gives196The next step is now letting $k \rightarrow \infty$ in the previous inequality, and for this we need to recall a few regularity properties of the functions $w_k$. An important point is that the way the functions $w_k$ have been defined gives that they belong to the Sobolev space $W^{1,p}(B_j)$ and this allows to apply the regularity theory available for such solutions. By (196) and the very definition of the functions $u_k$ (they are converging to $u$ in $W^{1,p-1}_0(\Omega )$) it follows that the sequence $\{w_k\}$ is bounded in $W^{1,p-1}(B_j)$; in turn this fact and the bound in (100) allows to get a uniform bound of the type $\Vert Dw_k\Vert _{L^\infty (\gamma B_j)}\le c (\gamma )$. In the same way we deduce, by using (101), that the sequence of maps $\{Dw_k\}$ is equicontinuous in $\gamma B_j$. We can therefore invoke Ascoli–Arzelá’s theorem that, together with a standard diagonal argument (recall that here $\gamma <1$ is arbitrary), gives that there exists a function $w \in W^{1,\infty }_\mathrm{loc}(B_j)\cap u + W^{p-1}_0(B_j)$ such that, up to a not relabelled subsequence, we have $w_k\rightarrow w$ and $Dw_k \rightarrow Dw$ locally uniformly in $B_j$. Now, the locally uniform convergence of the gradients $Dw_k$ allows to pass to the limits in the equations (195) and to conclude that $\hbox {div}\, a(x,Dw)=0$. Fatou’s lemma now givesand in the last estimate we have used a standard property of the weak$^*$-convergence of measures. A similar reasoning can be worked out with respect to (193) and (194). All in all we have proved the lemma with the choice $v_j\equiv w$. $\square$

With the previous lemma at our disposal the proofs of Theorems 10, 19 and 15 for SOLA proceed verbatim as in the case of standard energy solutions, with the sequence $v_j$ replacing everywhere the one in (112) whenever this is used. Indeed all the remaining facts used about the functions $v_j$ follow by Lemma 8. In particular, all the facts reported in Sects. 11 and 12.1 are still valid since the functions $v_j$ belong to $W^{1,p}_\mathrm{loc}(B_j)\cap W^{1,p-1}(B_j)$ and therefore standard regularity theory applies to them. The only difference is that the quantities $|\mu |(\bar{B}_j)$ must replace everywhere the analogous $|\mu |(B_j)$. This is clearly not a problem since the basic inequalities the quantities $|\mu |(\bar{B}_j)$ have to satisfy are implied by (116) and (179), which are already involving $|\mu |(\bar{B}_j)$. Needless to say, whenever we are considering vanishing limits as in (161) the same thing hold when considering closed balls.

## Theorem 3 as a corollary

The assertion of Theorem 3 can be now obtained as a corollary of the results presented up to now, and, by the approach in the previous section, directly for SOLA. Indeed, estimate (24) follows directly by estimate (40) and the fact that the finiteness of $\mathbf{I}_1^\mu (x,R)$ implies that $x$ is a Lebesgue point of $Du$. This is ensured by Theorem 15, see also Remark 1. It remains to prove that (25) implies the continuity of $Du$. For this we shall essentially revisit the proof of Theorem 15 given in the previous section and we shall see that the Cauchy sequence information in (170) holds locally uniformly in $x$. This means that the radius $r_\varepsilon$ can be chosen independently of the point $x$ as long as this varies in a fixed open subset $\Omega ' \Subset \Omega$. The continuity of $Du$ then follows by showing that it can be obtained as the locally uniform limit of the net of continuous maps defined by

197 $$\begin{aligned} x \rightarrow (Du)_{B(x,\varrho )} \qquad \hbox {for} \quad 0<\varrho \le d := \hbox { dist } (\Omega ', \partial \Omega )\,, \end{aligned}$$

which are obviously continuous. Note that we already know that the above maps converge pointwise to $Du$ (that is, they converges to its precise representative at every point) as established in Theorem 15. Now, a standard covering argument and the assumption (25) imply that $Du$ is locally bounded in $\Omega$. Therefore, selecting another open subset $\Omega ''$ such that $\Omega ' \Subset \Omega '' \Subset \Omega$, we set

198 $$\begin{aligned} \lambda _M:= c \Vert \mathbf{I}_1^{\mu }(\cdot , R)\Vert _{L^\infty (\Omega '')}^{1/(p-1)}+c\Vert |Du|+s\Vert _{L^\infty (\Omega '')} \end{aligned}$$

for some $R>0$, where $c$ is the constant appearing in (40). This number is going to replace the analogous one picked in (169), making it independent of $x$. Then, we observe that assuming (25) allows to satisfy condition (174), uniformly in $x$ (with an argument for instance based on computations as in (115)) and this implies that (172) holds again locally uniformly in $x$ via Theorem 19. This, and the assumption (25), imply that we can replace (177) and (178) by the stronger conditions

$$\begin{aligned} \sup _{x \in \Omega '}\, \left[ \int _0^{2 R_l} \frac{|\mu |(B(x,\varrho ))}{\varrho ^{n-1}} \, \frac{d\varrho }{\varrho } \right] ^{1/(p-1)}\le \frac{\delta _1^{5n} \lambda _M\varepsilon }{10^8 c_1c_2c_d} \end{aligned}$$

and

$$\begin{aligned} \sup _{x \in \Omega '}\, \sup _{0<\varrho \le R_l} \, E(Du, B(x,\varrho )) \le \frac{ \delta _1^{5n}\lambda _M\varepsilon }{10^8} \end{aligned}$$

respectively, where now in addition we take $R_l< \mathrm{dist}(\Omega ', \partial \Omega '')$ and $\lambda _M$ is defined as in (198). With the inequalities of the last two displays being now in force it is easy to check that all the inequalities after (179) become uniform with respect to the point $x$ considered. In particular, the numbers $\delta _1$ and $R_l$ that determine $r_\varepsilon$ via (181), that is the radius for which (170) holds, are independent of $x$. This means that the maps defined in (197) converge uniformly to $Du$ and the continuity of $Du$ therefore follows.

## Proof of Theorem 9

Theorem 9 has been again proved in a slightly different form in [40]. The proof of Theorem 9 goes essentially in two stages: we first obtain a pointwise estimate for a quantity like $|Du-G|$ (for any vector $G \in {\mathbb { R}}^n$). Dimensionally speaking, this quantity represents an oscillation of the gradient when $G$ is suitably chosen, i.e. when for instance is taken to be an average of the gradient. Then we conclude applying the sharp maximal inequality of Theorem 12 that in fact allows to estimate, in an integral way, oscillations of the gradient. Notice that in the rest of the proof we shall assume that

199 $$\begin{aligned} \mathbf{W}_{1-\frac{(1+\alpha ) (p-1)}{p},p}^\mu (x,R)+\mathbf{W}_{1-\frac{(1+\alpha ) (p-1)}{p},p}^\mu (y,R)< \infty \end{aligned}$$

holds, otherwise there is nothing to prove. We notice that this condition allows to conclude that both $x$ and $y$ are Lebesgue points of the gradient $Du$; this fact is indeed implied by Theorem 15, as the Wolff potentials appearing in (199) control the Riesz potential $\mathbf{I}_1^{\mu }$ at the points $x,y$ via a computation similar to the one in (26). We will anyway give a shorter proof of this fact to make the proof of Theorem 9 self-contained; see Lemma 9 below.

### General setting

We readopt the general setting of Theorem 10. Therefore we start by $x, y \in B_{R/4}$ and we select $r< R/2$ to be determined later. Then we follow the scheme described in Sect. 11. In particular, we define the shrinking balls $\{B_i\}$ as in (81); they are all centred at $x$, while eventually all the arguments will be reapplied when all the balls will be centred at $y$. The functions $v_j$ are defined in (82) while as usual we denoteThe number $\delta _1$ determining the shrinking rate of the balls $B_j$ is instead defined as

200 $$\begin{aligned} \delta _1 := \left( \frac{1}{10^7c_d}\right) ^{1/\beta }, \end{aligned}$$

where the constants $c_d$ and $\beta$ are from Theorem 17. This in turn yields a dependence of $\delta _1$ on $n,p,\nu , L$ only.

### Estimates on the excess

By the choice of $\delta _1$ we can apply Lemma 4 with $\alpha =1$; this gives

201 $$\begin{aligned} E_{j+1} \le \frac{1}{10^6} E_{j} +8c_1c_d\delta _1^{-n}\left[ \frac{|\mu |(B_{j})}{ r_{j}^{n-1}} \right] ^{1/(p-1)} \end{aligned}$$

for every $j \ge 0$. By summing inequalities (201) for $j \in \{k,\ldots ,h\}$, where $1\le k\le h$ are two arbitrary nonnegative integers, we gain

$$\begin{aligned} \sum _{j=k+1}^{h+1}E_{j} \le \frac{1}{10^6} \sum _{j=k}^{h}E_{j} +c\sum _{j=k}^{\infty }\left[ \frac{|\mu |(B_{j})}{ r_{j}^{n-1}} \right] ^{1/(p-1)}. \end{aligned}$$

so that

$$\begin{aligned} \sum _{j=k+1}^{h+1}E_{j} \le E_{k} +c\sum _{j=k}^{\infty }\left[ \frac{|\mu |(B_{j})}{ r_{j}^{n-1}} \right] ^{1/(p-1)} \end{aligned}$$

and, ultimately

$$\begin{aligned} \sum _{j=k}^{h+1}E_{j} \le 2E_{k} +c\sum _{j=k}^{\infty }\left[ \frac{|\mu |(B_{j})}{ r_{j}^{n-1}} \right] ^{1/(p-1)}\,, \end{aligned}$$

where $c \equiv c (n,p,\nu , L)$. By noticing that

$$\begin{aligned} \left[ \frac{|\mu |(B_{j})}{ r_{j}^{n-1}} \right] ^{1/(p-1)}= r_j^\alpha \left[ \frac{|\mu |(B_{j})}{ r_{j}^{n-1+\alpha (p-1)}} \right] ^{1/(p-1)}\le r^\alpha \left[ \frac{|\mu |(B_{j})}{ r_{j}^{n-1+\alpha (p-1)}} \right] ^{1/(p-1)} \end{aligned}$$

we conclude with

202 $$\begin{aligned} \sum _{j=k}^{h+1}E_{j} \le 2E_{k} +cr^\alpha \sum _{j=k}^{\infty }\left[ \frac{|\mu |(B_{j})}{ r_{j}^{n-1+\alpha (p-1)}} \right] ^{1/(p-1)}. \end{aligned}$$

Next, we estimate the last sum in terms of Wolff potentials as follows:

203 $$\begin{aligned} \mathbf{W}_{1-\frac{(1+\alpha ) (p-1)}{p},p}^\mu (x,2r)&= \int _0^{2r}\left[ \frac{|\mu |(B(x,\varrho ))}{ \varrho ^{n-1+\alpha (p-1)}} \right] ^{1/(p-1)}\, \frac{d\varrho }{\varrho }\nonumber \\&= \sum _{j=0}^\infty \,\,\, \int _{r_{j+1}}^{r_j} \left[ \frac{|\mu |(B(x,\varrho ))}{ \varrho ^{n-1+\alpha (p-1)}} \right] ^{1/(p-1)} \, \frac{d\varrho }{\varrho }\nonumber \\&\quad + \int _{r}^{2r} \left[ \frac{|\mu |(B(x,\varrho ))}{ \varrho ^{n-1+\alpha (p-1)}} \right] ^{1/(p-1)}\, \frac{d\varrho }{\varrho } \nonumber \\&\ge \sum _{j=0}^\infty \left[ \frac{|\mu |(B_{j+1})}{r_j^{n-1+\alpha (p-1)}} \right] ^{1/(p-1)} \int _{r_{j+1}}^{r_j} \frac{d\varrho }{\varrho }\nonumber \\&\quad + \left[ \frac{|\mu |(B_{0})}{(2r)^{n-1+\alpha (p-1)}} \right] ^{1/(p-1)} \int _{r}^{2r} \frac{d\varrho }{\varrho } \nonumber \\&\ge \delta _1^{(n-1)/(p-1)+\alpha } \log \left( \frac{1}{\delta _1}\right) \sum _{j=0}^\infty \left[ \frac{|\mu |(B_{j+1})}{r_{j+1}^{n-1+\alpha (p-1)}} \right] ^{1/(p-1)} \nonumber \\&\quad + \frac{ \log 2}{2^{(n-1)/(p-1)+\alpha }}\left[ \frac{|\mu |(B_{0})}{r_0^{n-1+\alpha (p-1)}} \right] ^{1/(p-1)} \nonumber \\&\ge \delta _1^{n} \sum _{j=0}^\infty \left[ \frac{|\mu |(B_{j})}{ r_{j}^{n-1+\alpha (p-1)}}\right] ^{1/(p-1)}. \end{aligned}$$

Now, by taking $k=0$ and $h=i$ in (202) we have

$$\begin{aligned} \sum _{j=0}^{i+1}E_{j} \le 2E_{0} +cr^\alpha \sum _{j=0}^{\infty }\left[ \frac{|\mu |(B_{j})}{ r_{j}^{n-1+\alpha (p-1)}} \right] ^{1/(p-1)} \end{aligned}$$

and merging the content of the last two displays we conclude with

204 $$\begin{aligned} \sum _{j=0}^{i+1}E_{j} \le 2E_{0} +cr^\alpha \mathbf{W}_{1-\frac{(1+\alpha ) (p-1)}{p},p}^\mu (x,R) \end{aligned}$$

where we have used the fact that $2r\le R$ and the constant $c$ depends only on $n,p,\nu , L$ thanks the choice of $\delta _1$ made in (200). In particular, again taking $\alpha =0$ and eventually letting $i \rightarrow \infty$ in the previous inequality gives

205 $$\begin{aligned} \sum _{j=0}^{\infty }E_{j} \le 2E_{0} +c\mathbf{W}_{1/p,p}^\mu (x,R). \end{aligned}$$

### Pointwise estimate and Lebesgue point criterion

Let us prove

#### **Lemma 9**

(Wolff potentials control Lebesgue points) The condition

206 $$\begin{aligned} \mathbf{W}_{1/p,p}^\mu (x,R) < \infty \end{aligned}$$

implies that the following limit exists:

$$\begin{aligned} \lim _{i \rightarrow \infty }\, (Du)_{B_i}=Du(x). \end{aligned}$$

#### *Proof*

Observe that exactly as in (140) we have, whenever $h> k$

207 $$\begin{aligned} |(Du)_{B_{h}} - (Du)_{B_{k}}| \le \delta _1^{-n}\sum _{j=k}^{h-1} E_{j}. \end{aligned}$$

Using (202) with $\alpha =0$ we gain

208 $$\begin{aligned} |(Du)_{B_{h}} - (Du)_{B_{k}}| \le c E_{k} +c\sum _{j=k}^{\infty }\left[ \frac{|\mu |(B_{j})}{ r_{j}^{n-1}} \right] ^{1/(p-1)}. \end{aligned}$$

Now, notice that (203) with $\alpha =0$ gives

209 $$\begin{aligned} \sum _{j=0}^{\infty }\left[ \frac{|\mu |(B_{j})}{ r_{j}^{n-1}} \right] ^{1/(p-1)} \le c\mathbf{W}_{1/p,p}^\mu (x,2r) < \infty \end{aligned}$$

and this, together with (205) gives

$$\begin{aligned} \lim _{k \rightarrow \infty }\, E_{k} +\sum _{j=k}^{\infty }\left[ \frac{|\mu |(B_{j})}{ r_{j}^{n-1}} \right] ^{1/(p-1)}=0. \end{aligned}$$

In turn this last fact and (208) imply that $\{(Du)_{B_i}\}$ is a Cauchy sequence and the assertion is proved. Notice that a slight variant of this argument (see for instance Sect. 14.2) leads to establish that $x$ is a Lebesgue point of $Du$ in the usual sense. See also Sect. 17 below. $\square$

As (199) implies the finiteness condition in (206) the previous lemma implies in particular that

210 $$\begin{aligned} \lim _{i \rightarrow \infty }|(Du)_{B_{i}}-G|=|Du(x)-G| \end{aligned}$$

holds whenever $G \in {\mathbb { R}}^n$. Let us use (140) for the choice $k_1=0$ and $k_2=i\ge 1$ to get

$$\begin{aligned} |(Du)_{B_{i}}-(Du)_{B_{0}}| \le \delta _1^{-n}\sum _{j=0}^{i-1} E_{j}\equiv c\sum _{j=0}^{i-1} E_{j} \end{aligned}$$

with $c\equiv c (n,p,\nu , L)$. Further use of triangle inequality then gives

$$\begin{aligned} |(Du)_{B_{i}}-G| \le |(Du)_{B_{0}}-G| + c\sum _{j=0}^{i-1} E_{j} \end{aligned}$$

so that, using (204) we obtain

$$\begin{aligned} |(Du)_{B_{i}}-G| \le |(Du)_{B_{0}}-G| + cE_{0} +cr^\alpha \mathbf{W}_{1-\frac{(1+\alpha ) (p-1)}{p},p}^\mu (x,R)\,, \end{aligned}$$

where the constant $c$ depends only on $n,p,\nu , L$. Letting $i \rightarrow \infty$ and recalling (210) we conclude with


### Use of the maximal estimate and conclusion

Writing inequality (211) for $$y$$ instead of $$x$$ and summing up gives211We recall that we can still choose $$G$$ and $$r\le R/2$$; we therefore take $$ G:=(Du)_{B(x,3r)}$$ and $$ r:= |x-y|/2$$, so that $$ B(y,r)\subset B(x,3r)$$ and therefore, using also (98) we have212Now, notice that as we are assuming that the initial ball $$B_{R}$$ is such that $$x,y \in B_{R/4}$$, then we have necessarily $$|x-y|\le R/2$$ so that now $$r\le R/4$$ and $$B(x,3r)\subset B(x,3R/4)\subset B_{R}$$. Therefore we use the definition of sharp maximal operator and Theorem 12 to estimateIt is possible to estimate the fractional maximal operators by Wolff potentials via completely elementary means; for instance, the following inequality is proved in [40, Lemma 4.1] and holds whenever $$\gamma \in (0,1)$$:

213 $$\begin{aligned} \left[ M_{\beta ,\gamma R}(\mu )(x)\right] ^{1/(p-1)} \le \frac{\max \{\gamma ^{(\beta -n)/(p-1)},1\}}{(-\log \gamma ) |B(0,1)|^{1/(p-1)} } \mathbf{W}_{\beta /p,p}^\mu (x,R). \end{aligned}$$

The content of the last two displays and (98) yieldsConnecting the last inequality to (212) and eventually to (211) completes the proof of Theorem 9.

## Proof of Theorem 16

The proof goes in two steps, reported in Sects. 17.1 and 17.2, respectively. We here give the whole proof of Theorem 16 in the case of an energy solution $u \in W^{1,p}(\Omega )$; the case of a general SOLA can be treated adapting the arguments valid for the energy case along the lines described in Sect. 14.4 for Theorem 15. In the rest of the proof all the balls will be centred at the point $x$, starting from an initial ball $B(x,R) \subset \Omega$ with $R\le 1$, which is not restrictive of course.

### Smallness of the excess

Here we prove214that is, we prove that for every $\tilde{\varepsilon }>0$ there exists $\tilde{R}>0$, basically depending on $\tilde{\varepsilon }$ and on the point $x$, such that

215 $$\begin{aligned} \varrho \le \tilde{R} \Longrightarrow E(u, B(x,\varrho ))< \tilde{\varepsilon }. \end{aligned}$$

To start with, we notice that by using (44) with $\alpha =0$, together with (213) with $\beta =p$ to establish the finiteness of the relevant maximal operator, we get216Thanks to this we are able to determine $\varepsilon >0$ small enough in order to have217With $\varepsilon$ been determined, we define $\delta _1$ as

218 $$\begin{aligned} \delta _1 := \left( \frac{ \varepsilon }{10^8\tilde{c}_d}\right) ^{1/\tilde{\beta }}, \end{aligned}$$

where the constants $\tilde{c}_d$ and $\tilde{\beta }$ are from Theorem 18. In turn, having $\delta _1$ being defined, we choose the radius $r \le R/2$ such that the smallness condition

219 $$\begin{aligned} 16\tilde{c}_1\tilde{c}_d\delta _1^{-3n}\mathbf{W}_{1,p}^\mu (x, 2r) \le \frac{\tilde{\varepsilon }}{2} \end{aligned}$$

is satisfied. The constant $\tilde{c}_1$ has been introduced in (77). Notice that this is possible since the assumption in (62) and the definition of Wolff potential imply that

220 $$\begin{aligned} \lim _{\varrho \rightarrow 0}\, \mathbf{W}_{1,p}^\mu (x,\varrho )=0. \end{aligned}$$

Determining $r$ allows to consider the sequence of shrinking balls $\{B_j\}$ as defined in (81); the functions $\{v_j\}$ are as in (112). Accordingly, we defineFinally, an argument similar to the ones in (203) yields

221 $$\begin{aligned} \delta _1^{n} \sum _{j=0}^\infty \left[ \frac{|\mu |( B_{j})}{ r_{j}^{n-p}}\right] ^{1/(p-1)} \le \mathbf{W}_{1,p}^\mu (x,2r). \end{aligned}$$

This is indeed an inequality that actually works whenever $\delta _1 \in (0,1/4)$ is a free parameter and the balls $\{B_j\}$ are defined as $B_j := B(x,\delta _1^jr)$.

Now, the first thing we do is looking at Lemma 4 (when $\alpha =0$) and make computations similar to the one involved there, but using Theorem 18 instead of Theorem 17, and using (77) instead of (76). Specifically, for every $j \ge 0$ we haveRecalling (218) we conclude that the following estimate holds for every $j \ge 0$:

222 $$\begin{aligned} \tilde{E}_{j+1} \le \frac{ \varepsilon }{10^6} \tilde{E}_{j} +8\tilde{c}_1\tilde{c}_d\delta _1^{-n}\left[ \frac{|\mu |(B_{j})}{ r_{j}^{n-p}} \right] ^{1/(p-1)}+ \tilde{\varepsilon }r_js. \end{aligned}$$

Summing up the preceding inequalities and proceeding as in Sect. 16.2 yields

223 $$\begin{aligned} \sum _{j=k}^{h}\tilde{E}_{j} \le \varepsilon \tilde{E}_{k-1} + \varepsilon rs\sum _{j=0}^{\infty }\delta _1^j + 16\tilde{c}_1\tilde{c}_d\delta _1^{-n}\sum _{j=0}^{\infty }\left[ \frac{|\mu |(B_{j})}{ r_{j}^{n-p}} \right] ^{1/(p-1)}, \end{aligned}$$

which is valid whenever $1\le k\le h$ are integers. We then use (217) to estimate the first two terms in the right hand side of the previous inequality and (219)–(221) to estimate the remaining one; therefore we conclude with

224 $$\begin{aligned} \sum _{j=1}^{\infty }\tilde{E}_{j} \le \tilde{\varepsilon }. \end{aligned}$$

This last inequality in particular implies that225On the other hand, take now $\varrho ' \le r_{1}= \delta _1r$ and determine $k\ge 1$ such that $r_{k+1}< \varrho \le r_{k}$. This means that $\varrho ' = \delta _1^k r'$ for $r' \in (\delta _1r, r)$. Now, in the proof of (225) we replace $r$ by $r'$ and we get a new chain of cylinders $B_{j}'=B(x, \delta ^jr')$ to which we apply the same reasoning as above; then (225) follows for $\varrho = r'_j$ and every $j\ge 1$. In particular it follows that $E(u,B(x,\varrho '))\le \tilde{\varepsilon }$ and this means now that now (215) holds for $\tilde{R} = \delta _1r$ where $r$ has been determined via the choice in (219).

### Proof that $x$ is a Lebesgue point of $u$

We are now ready for the proof of (63), therefore showing that for every $\varepsilon >0$, there exists a radius $r_\varepsilon$, depending on $\varepsilon$, such that

226 $$\begin{aligned} |(u)_{B(x,\varrho )}-(u)_{B(x,\tau )}|\le \varepsilon \qquad \hbox {holds whenever} \ 0< \tau < \varrho \le r_\varepsilon . \end{aligned}$$

We now define

227 $$\begin{aligned} \delta _1 := \left( \frac{1}{10^8\tilde{c}_d}\right) ^{1/\tilde{\beta }} \end{aligned}$$

where $\tilde{c}_d$ appears in (106), and then we take a radius $\tilde{R}_l$ such that

228 $$\begin{aligned} \sup _{0<\varrho \le \tilde{R}_l} \, E(u, B(x, \varrho ))+16\tilde{c}_1\tilde{c}_d\delta _1^{-2n}\mathbf{W}_{1,p}^\mu (x, 2\tilde{R}_l) + 2s\tilde{R}_l\le \frac{\delta _1^n\varepsilon }{3}. \end{aligned}$$

We notice that this is possible thanks to (214) and (220). With $\delta _1$ and $\tilde{R}_l$ having been defined, we then use the usual shrinking chain of balls $\{B_j\}$ defined in (81) with $r=\tilde{R}_l$, so that, in particular we have $r_j=\delta _1^j\tilde{R}_l$; again, the functions $\{v_j\}$ are as in (112). Similarly to (207), whenever $h > k \ge 1$ are integers it holds that

229 $$\begin{aligned} |(u)_{B_{h}} - (u)_{B_{k}}| \le \delta _1^{-n}\sum _{j=k}^{h-1} \tilde{E}_{j}. \end{aligned}$$

We now observe that (223) still works in this setting with $\varepsilon =1$, according to the choice in (227). This is

$$\begin{aligned} \sum _{j=k}^{h}\tilde{E}_{j} \le \tilde{E}_{k-1} + rs\sum _{j=0}^{\infty }\delta _1^j + 16\tilde{c}_1\tilde{c}_d\delta _1^{-2n} \mathbf{W}_{1,p}^\mu (x,2r), \end{aligned}$$

and we also used (221), which is again an inequality that whenever $\delta _1 \in (0,1/4)$ is a fixed parameter. Combining the inequalities in the last two displays together with (228), we obtain that

230 $$\begin{aligned} |(u)_{B_{h}} - (u)_{B_{k}}|\le \frac{\varepsilon }{3}\qquad \hbox {holds for every choice of } \ h > k \ge 1. \end{aligned}$$

We can now prove (226) with the choice $r_\varepsilon :=\delta _1 \tilde{R}_l.$ Indeed, let us fix $0 < \tau < \varrho \le r_\varepsilon$. This means that there exist two integers $k$ and $h$, such that $1 \le k \le h$,

$$\begin{aligned} \delta _1^{k+1}\tilde{R}_l < \varrho \le \delta _1^{k}\tilde{R}_l \qquad \hbox {and} \qquad \delta _1^{h+1}\tilde{R}_l < \tau \le \delta _1^{h}\tilde{R}_l \end{aligned}$$

hold. Applying (228) yieldsand, similarly,

$$\begin{aligned} |(u)_{B(x, \tau )}- (u)_{B_{h+1}}|\le \frac{\varepsilon }{3}. \end{aligned}$$

The last two inequalities and (230) establish (226) and the proof of Theorem 16 is complete.

## Back to the roots: Theorem 1 (sketch)

The arguments developed for Theorem 16 allow to get a quick proof of Theorem 1, that we sketch in the following lines. We repeat the construction of Sects. 17.1– with $\delta _1$ as in (227), but starting from any ball $B(x,R) \subset \Omega$, with $r =R/2$; the sequence $\{B_j\}$ is once again accordingly defined as in (81). In (229) we take $k=1$ and let $h \rightarrow \infty$; this yields

$$\begin{aligned} |u(x) - (u)_{B_{0}}| \le \delta _1^{-n}\sum _{j=1}^{\infty } \tilde{E}_{j}. \end{aligned}$$

Using this last inequality together with (221) and (223) (here it is $\varepsilon =1$) yieldsfor a constant $c \equiv c (n,p,\nu , L)$, that is (20). The proof of the continuity of $u$ under assumption (21) follows instead making the arguments for the proof of Theorem 16 uniform with respect to $x$, in the same way we have done in Sect. 15 with respect to the proof of Theorem 15.
